# Supplementary material for: “Of course, drones delivering urgent medicines are necessary. But I would not use them until…” Insights from a qualitative study on users’ needs and requirements regarding the use of medical drones
Source: PLoS One. 2023 May 8;18(5):e0285393. doi: 10.1371/journal.pone.0285393 (PMC10166545; doi:10.1371/journal.pone.0285393)
Supplement: S1 Appendix — (DOCX) [file pone.0285393.s001.docx]

**S1. ADApp Design**

The concept of the pharmacy-drone-study (Apotheken-Drohnen-App; ADApp) is to combine a pharmacy app with drone logistics for the delivery of urgent medicines or contactless delivery for comprehensive medication supply, mainly in rural areas. The ADApp is a novelty for the German region mentioned above. Currently, drones are already being tested for health care logistics between clinics in large cities (e.g., Munich [1]) or used for health care in rural areas outside of Europe (e.g., in Africa [2–5]). Special about ADApp is that, for the first time, the entire supply process can be coordinated digitally, from the issuing of the e-prescription at the general practitioner, to the order at the pharmacy, to delivery by drone directly to the patient’s doorstep or to an institution. We consider after the current common proof of concepts flights the legally, administrative and logistically integration of drones within the continuous supply process as the next important step to reach practical implementation. The aim of the project is to design an app and drone system in a way that increases usability, acceptability, and satisfaction of basic psychological needs in order to evaluate the effectiveness of using app-assisted drone-based medicine delivery. The feasibility study is using a phased user-centered design to explore the needs and requirements of users as well as the practical and logistical facilitators and challenges involved in the development and implementation of such technology. Users’ involvement at every developmental step will ensure the feasibility of medical drone delivery (see **Figure 1**).

The ADApp project design is embedded in a broader context of user-centred framework oriented at Farao’s and colleagues modified *Information System Research framework incorporating modes of design thinking* [6]. The classical *Information System Research framework (ISR)* uses three research cycles, where the environment of users is understood (relevance cycle), objects relating to the problem are created (design cycle), and findings from the evaluation are combined with the existing knowledge base (rigor cycle) [7]. An example that has been used in ISR is the technology acceptance model (TAM; [8,9]), which shows that better usability and higher usefulness of a technology lead to a higher acceptance of the same. The *Design Thinking (DT)* approach is used to prioritize the needs of users to solve complex problems [10,11]. It includes different steps. It starts with the identification of needs with the initial understanding of the users and the problem itself (*empathize*). The user feedback is afterwards analyzed into identifiable needs (*define*). The next mode is the *ideation*, which involves generating diverse ideas that in the next step are translated from thoughts to physical representations (*prototyping*). The next mode is *testing.* Its purpose is to refine and improve prototypes as well as simulating the use in the context. In a final step (*implementing*), the results are evaluated in medium-term and long-term change which can result in the first step, the identification of needs (Altman, 2018). Taken together, ISR starts from typical, observed problems in practice and solves them with scientific principles at a high level of abstraction. DT identifies additional aspects of the design process by focusing on the needs and requirements of users [12]. Thus, Farao et al. [6] combined these two approaches, which can be used for desigining and re-designing technologies. This approach (1) involves the environment (ISR: relevance cycle) as well as problems, needs, and requirements of users and companies (DT: empathize and define mode); (2) promotes the transferability of the design knowledge (ISR: rigor cycle) through scientific theories and models (e.g., the technology acceptance model), results of existing artefacts (e.g., literature reviews), and through testing (DT), which includes the engagement of users (e.g., evaluation of functionalities); (3) centers the users throughout the design process by users involvement during technical development (ISR: design cycle, DT: ideation and prototyping), which in fact contributes to the knowledge base and involves the needs of users at all times (see **Figure 1**).


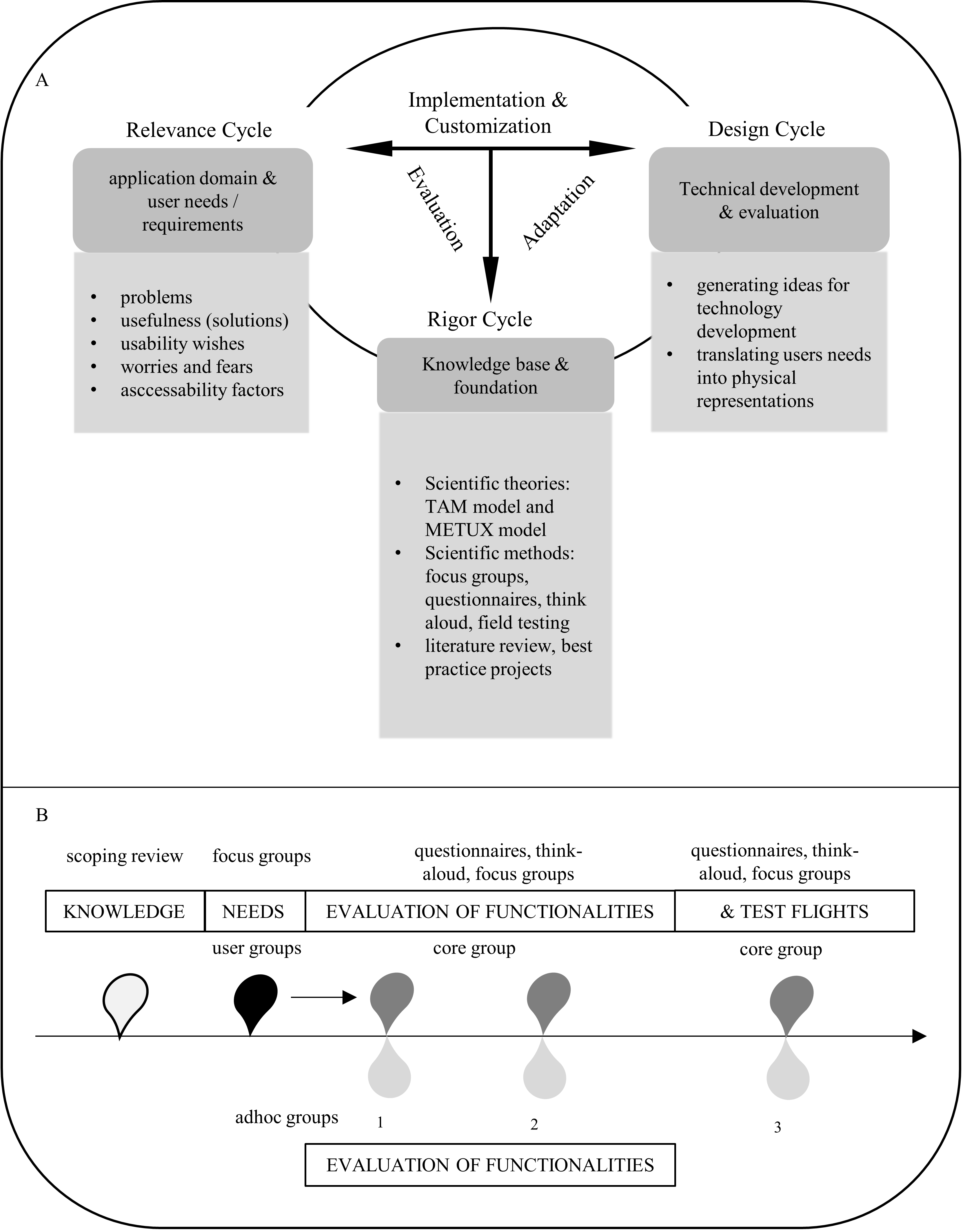


**Figure 1.** **A.** **Users’ involvement at every developmental process.**

Users’ needs are scientifically assessed within the relevance cycle and evaluated within the rigor cycle which contributes to the knowledge base. The knowledge base contributes to the adaptation of technical development based on the translation of scientific findings into technical ideas. Users’ needs are incorporated into technical development and technical development is adapted to users and the knowledge base within the design cycle. Technical development is tested and evaluated with users within the relevance cycle.

**B. The whole ADApp study design.**

Based on the cyclic and iterative nature of the user centered approach, at all stages the users’ needs, technical development, and knowledge base incorporate all conditions of the cycles seen in figure 1 A. Nevertheless, the study started with a literature review about the human drone interaction in the delivery of medical supplies. Scientific theories, such as the METUX model (Peters et al. 2018) or TAM model [8,9], contribute to the knowledge base which were important for choosing the study design. Next, focus groups (FG) were conducted that aimed at collecting the problems, needs, and requirements users have if they imagine using such technology (the present study). Four user groups were defined: general practitioners, pharmacists, nurses, and SAR-CoV2-infected patients. In this step, it was essential that the user’s needs are translated into a set of functional requirements and design guidelines. Thus, potential themes were identified at the basis of transcriptions and coding of text segments of FG. These themes are relevant to the software system goals. Thus, prototypes can be sketched for eliciting feedback form users. This is essential for a deeper understanding of the intended goal of an app or technology. A core group will be established, consisting partly of participants of the FG. With the help of walkthroughs and usability testing, the technology will be evaluated and refined iteratively through three evaluation circles. Walkthroughs will be conducted in that participants are encouraged to think aloud and provide feedback on the difficulties and facilitators during the experiencing of the app and drone. It aims at ensuring that the performance of the app and drone matches the user requirements. These walkthroughs will be analyzed qualitatively. For assessing the usability, acceptability, and the satisfaction of psychological needs, the users answer a set of questionnaires (TUI, SUS, METUX-questionnaires). These questionnaires will be analyzed quantitatively. At this step, three different adhoc groups will be established at each walkthrough which act as control groups. We consider this controlled approach as important to deal with potential biasing effect due to response shift through multiple feedback cycles [13]. In a last step, participants will engage with the app and drone in a natural environment (airport) by aiming at assessing how the participants interact with the technology. Usability and acceptability will be assessed qualitatively with discussions and quantitatively with questionnaires [14]. METUX: Motivation, Engagement, Thriving in User Experience; TAM: Technology Acceptance Model; TUI: Technology Usage Inventory; SUS: Usability Scale Assessments.

**References**

**1**. Ärzteblatt.de. Automatisierte Medikamenten­transporte per Drohne [updated 2021; cited 18 May 2021]. Available from: https://www.aerzteblatt.de/nachrichten/122802/Automatisierte-Medikamententransporte-per-Drohne.

**2**. Kelland K. Drones to deliver vaccines, blood and drugs across Ghana. Reuters 2019 [cited 18 May 2021]. Available from: https://www.reuters.com/article/us-health-vaccines-drones-idUSKCN1S0175.

**3**. Drones could speed up HIV tests in remote areas. Reuters 2016 [cited 18 May 2021]. Available from: https://www.reuters.com/article/us-malawi-hiv-drones-idUSKCN0XH1ZN.

**4**. Werber C. Rwanda Is Using Drones To Deliver Blood Donations To Remote Health Centers. The Huffington Post 2016 [cited 18 May 2021]. Available from: https://www.huffpost.com/entry/drone-delivery-service-launched-in-rwanda_n_5800f353e4b0162c043b7739.

**5**. Africa’s first humanitarian drone testing corridor. UNICEF 2017 [cited 18 May 2021]. Available from: https://www.unicef.org/innovation/drones/africa-first-humanitarian-drone-corridor-malawi.

**6**. Farao J, Malila B, Conrad N, Mutsvangwa T, Rangaka MX, Douglas TS. A user-centred design framework for mHealth. PloS One. 2020; 15:e0237910. doi: 10.1371/journal.pone.0237910 PMID: 32813711.

**7**. Hevner A. A Three Cycle View of Design Science Research. Scandinavian Journal of Information Systems. 2007; 19. Available from: https://aisel.aisnet.org/sjis/vol19/iss2/4.

**8**. Kothgassner OD, Felnhofer A, Hauk N, Kastenhofer E, Gomm J, Ryspin-Exner I. TUI: Technology Usage Inventory: Vienna: FFG; 2012.

**9**. Davis FD. Perceived Usefulness, Perceived Ease of Use, and User Acceptance of Information Technology. MIS Quarterly. 1989; 13:319. doi: 10.2307/249008.

**10**. Altman M. Design Thinking in Health Care. Preventing Chronic Disease. 2018; 15. doi: 10.5888/pcd15.180128.

**11**. Roberts JP, Fisher TR, Trowbridge MJ, Bent C. A design thinking framework for healthcare management and innovation. Healthcare. 2016; 4:11–4. doi: 10.1016/j.hjdsi.2015.12.002.

**12**. Robra-Bissantz S, Strahringer S. Wirtschaftsinformatik-Forschung für die Praxis. HMD. 2020; 57:162–88. doi: 10.1365/s40702-020-00603-0.

**13**. Oort FJ, Visser MRM, Sprangers MAG. Formal definitions of measurement bias and explanation bias clarify measurement and conceptual perspectives on response shift. J Clin Epidemiol. 2009; 62:1126–37. Epub 2009/06/21. doi: 10.1016/j.jclinepi.2009.03.013 PMID: 19540722.

**14**. McCurdie T, Taneva S, Casselman M, Yeung M, McDaniel C, Ho W, et al. mHealth Consumer Apps: The Case for User-Centered Design. Biomedical Instrumentation & Technology. 2012; 46:49–56. doi: 10.2345/0899-8205-46.s2.49.
