# Supplementary material for: “Of course, drones delivering urgent medicines are necessary. But I would not use them until…” Insights from a qualitative study on users’ needs and requirements regarding the use of medical drones
Source: PLoS One. 2023 May 8;18(5):e0285393. doi: 10.1371/journal.pone.0285393 (PMC10166545; doi:10.1371/journal.pone.0285393)
Supplement: S2 Appendix — (DOCX) [file pone.0285393.s002.docx]

**S2 Table.** COREQ Checklist.

| **No** | **Item** | **Guide questions** | **Answer** | **Location where item is reported** |
| --- | --- | --- | --- | --- |
| **Domain 1: Research term and reflexivity** | | | |  |
| *Personal Characteristics* | | | |  |
| 1. | Interviewer/facilitator | Which author/s conducted the interview or focus group? | M1: FS  M2: DP or PJ | 1 |
| 2. | Credentials | What were the researcher’s credentials? | PhD | 1 |
| 3. | Occupation | What was their occupation at the time of the study? | research associate, Head of Working Group | 1 |
| 4. | Gender | Was the researcher male or female? | FS: female  DP and PJ: male | 1 |
| 5. | Experience and training | What experience or training did the researcher have? | FS: experience in mixed-method studies but not special in focus groups  DP: experience in qualitative methods, especially in focus groups  PJ: experience in mixed-method studies and also in focus groups | 1 |
| *Relationship with participants* | | | |  |
| 6. | Relationship established | Was a relationship established prior to study commencement? | no | 35 |
| 7. | Participant knowledge of the interviewer | What did the participants know about the researcher? | aim and reasons for doing the research | 8 |
| 8. | Interviewer characteristics | What characteristics were reported about the interviewer/facilitator? | name, researchers credentials, position in project | 7 |
| **Domain 2: study design** | | | |  |
| *Theoretical framework* | | | |  |
| 9. | Methodological orientation and Theory | What methodological orientation was stated to underpin the study? | user-centered framework, focus groups, content analysis | 6-10 |
| *Participant selection* | | | |  |
| 10. | Sampling | How were participants selected? | purposive, snowball | 6 |
| 11. | Method of approach | How were participants approached? | telephone, email | 8 |
| 12. | Sample size | How many participants were in the study? | 36 participants | 11 |
| 13. | Non-participation | How many people refused to participate or dropped out? Reasons? | n/a | n/a |
| *Setting* | | | |  |
| 14. | Setting of data collection | Where was the data collected? | online via WebEx | 8 |
| 15. | Presence of non-participants | Was anyone else present besides the participants and researchers? | no | 8 |
| 16. | Description of sample | What are the important characteristics of the sample? | gender, age, role characteristics | 11 |
| *Data Collection* | | | |  |
| 17. | Interview guide | Were questions, prompts, guides provided by the authors? Was it pilot tested? | Questions were provided by authors; no pilot testing was conducted but focus group instrument was peer-group developed | 7, S3 |
| 18. | Repeat interviews | Were repeat interviews carried out? If yes, how many? | no | 8,33 |
| 19. | Audio/visual recording | Did the research use audio or visual recording to collect the data? | audio recording | 9 |
| 20. | Field notes | Were field notes made during and/or after the interview or focus group? | yes | 9 |
| 21. | Duration | What was the duration of the interviews or focus group? | 60-90 minutes | 8 |
| 22. | Data saturation | Was data saturation discussed? |  | n/a |
| 23. | Transcripts returned | Were transcripts returned to participants for comment and/or correction? | no, but field notes were made available for participants for comments and corrections | 8 |
| **Domain 3: analysis and findings** | | | |  |
| *Data analysis* | | | |  |
| 24. | Number of data coders | How many data coders coded the data? | two (FS and DP) | 8-9 |
| 25. | Description of the coding tree | Did authors provide a description of the coding tree? | yes; see Table 1 | 10 |
| 26. | Derivation of themes | Were themes identified in advance or derived from the data? | in advance | 10 |
| 27. | Software | What software, if applicable, was used to manage the data? | f4analyse 2 | 9 |
| 28. | Participant checking | Did participants provide feedback on the findings? | yes (see point 23) | 8 |
| *Reporting* | | | |  |
| 29. | Quotations presented | Were participant quotations presented to illustrate the themes / findings? Was each  quotation identified? | yes; identification according to role characteristics | 11-26 |
| 30. | Data and findings consistent | Was there consistency between the data presented and the findings? |  | n/a |
| 31. | Clarity of major themes | Were major themes clearly presented in the findings? | yes; subheadings according to themes; figures | 11-26 |
| 32. | Clarity of minor themes | Is there a description of diverse cases or discussion of minor themes? |  | n/a |

*From*: Tong, Allison; Sainsbury, Peter; Craig, Jonathan (2007): Consolidated criteria for reporting qualitative research (COREQ): a 32-item checklist for interviews and focus groups. In: International journal for quality in health care: journal of the International Society for Quality in Health Care 19 (6), S. 349–357. DOI: 10.1093/intqhc/mzm042
