# Supplementary material for: “Of course, drones delivering urgent medicines are necessary. But I would not use them until…” Insights from a qualitative study on users’ needs and requirements regarding the use of medical drones
Source: PLoS One. 2023 May 8;18(5):e0285393. doi: 10.1371/journal.pone.0285393 (PMC10166545; doi:10.1371/journal.pone.0285393)
Supplement: S3 Appendix — (DOCX) [file pone.0285393.s003.docx]

**S3. Interview Guide**

Opener:

M1: “Thank you very much for taking the time to discuss with us today the topic of a pharmacy drone app for securing the supply of people with and without care needs, especially in rural regions. In this way, you are making a contribution to the development of a contactless and nationwide supply of medicines. We invited you because you are all users of the app [patients, caregivers, relatives of patients in need of palliative care] / the system [pharmacists, physicians] and interact with the drone [pharmacists, patients, caregivers, relatives of patients in need of palliative care]. We would like to hear your opinions and needs from your perspective on this topic.

We will audio record this conversation. We need this for the evaluation afterwards. The audio will be deleted after the evaluation. At the beginning, we will each briefly introduce ourselves (M 1 and M 2 begin)."

Case study:

M1: “Imagine the following scenario: a patient with Covid-19 infection is in quarantine. Previous studies suggest that medication (e.g., with budesonide) in the early acute phase has positive effects on the course of recovery. The supply of this drug for inhalation is particularly suitable the earlier it is used. Any delay in taking the drug will affect its effectiveness. Since the patient is in quarantine, the quick and non-contact delivery of the drug is necessary. One solution could be a drone based medication delivery.

The general practitioner issues the patient an e-prescription for the inhalation medication. The patient gets the QR code sent to their app. The patient sends the QR code to a pharmacy that can offer drone delivery. The pharmacy staff processes the order. When the medication is ready for delivery, the patient gets a message on their app that the delivery will be delivered at a specific time.

Once the drone arrives at the patient's home, the patient receives a message on the app. After a confirmation that the patient is ready to receive the medication, the medication is delivered. The patient confirms receipt, and the drone flies back to the pharmacy.”

**S3 Table.** Interview guide questions assigned to category and TUI factors.

| **No.** | **category** | **TUI factor** | **Question** |
| --- | --- | --- | --- |
|  |  |  |  |
| 1 | problems | curiosity and interest | Where can you think of examples of people having problems getting care under pandemic conditions? |
|  |  |  |  |
| 2 | knowledge and competence | curiosity and interest | Do you have knowledge and competence in handling with medication apps? |
|  |  |  |  |
| 3 | knowledge and competence | curiosity and interest | Do you have knowledge and competence in handling with drones? |
|  |  |  |  |
| 4 | usefulness | usefulness | Would you use the pharmacy drone app? Why or why not?  What would it take for you to use this technique? |
|  |  |  |  |
| 5 | communication | usability | Do you like to have personal contact or communication options? At what point in the process would you like to have personal contact? And to whom? |
|  |  |  |  |
| 6 | delivery process | usability | How do you imagine the process in the delivery of drugs? Can you imagine it well?  What features needs the delivery process?  How would the process be as barrier-free as possible? |
|  |  |  |  |
| 7 | handover | usability | What can the physical transfer of the drug look like from your perspective? |
|  |  |  |  |
| 8 | accessibility | accessibility | What information channels do you use to learn about such technologies? |
|  |  |  |  |
| 9 | concerns | skepticism and fearfulness | Do you have any other suggestions or concerns you want to let us know? |
|  |  |  |  |
