# Supplementary material for: “Of course, drones delivering urgent medicines are necessary. But I would not use them until…” Insights from a qualitative study on users’ needs and requirements regarding the use of medical drones
Source: PLoS One. 2023 May 8;18(5):e0285393. doi: 10.1371/journal.pone.0285393 (PMC10166545; doi:10.1371/journal.pone.0285393)
Supplement: S4 Appendix — (DOCX) [file pone.0285393.s004.docx]

**S4. Qualitative content analysis – additional selection of in-depth examples**

**S4 Table.** Qualitative content analysis – additional selection

| **Category** | **In-depht examples** | | | |
| --- | --- | --- | --- | --- |
| **Participant** | **General practitioners** | **Pharmacists** | **Nurses** | **Patients** |
| Problems | General practitioners noted that independently of the pandemic situation, rush hours in cities caused delays in medication delivery, too: “*That's where our couriers take so long to get from Berlin-Mitte to Berlin-Wedding at rush hour.”* | *“[…] we have a lot of isolated solutions that are making more work overall.”* | *“Certain medicines were simply missing in the morning."* The nurses also stated out that *"[…] due to the pandemic, we had patients at the beginning who said we didn't want any visitors because they were afraid that we would bring Corona.”* | (All important quotes in the article) |
| General Usability | (All important quotes in the article) | *“When the e-prescription is forwarded to the pharmacy, that is fed in right away and the system knows: in stock, can be delivered immediately and is translated into the time I can tell the patient when to expect the delivery.”* | (All important quotes in the article) | (All important quotes in the article) |
| Communication Usability | Not all participants of the general practitioner’s group like to have immediate communication: *“And just have, if necessary, ways of asking back, what it looks like. For example, “I got the medication” - thumbs up. “I know how to use it” - thumbs up. “I tolerate it quite well” - thumbs up. Or just the possibility to say: “I got it, but I am not quite sure how to take it now. Please reply to me or call me or I'll call you right now.””* | (All important quotes in the article) | *“I can imagine that the app might also provide instructions on how to take the medication. I don't know if everyone in the outpatient care service is always familiar with all the medications, how they should perhaps be taken, and instructions perhaps also for the patients themselves.”* | *“But I think a quick answer is also important, especially when you are now in quarantine, a bit on your own and perhaps afraid. When you need a medicine, you're usually not well, and I think it's very important that you get an answer as quickly as possible.”* |
| Process Useability | *“Then I have a hook - e-prescription issued; e-prescription received by pharmacist; drone packed. And the patient acknowledges receipt afterwards and says: “got it.””* | *“[…] The pharmaceutical staff looks over it again. If all the information is correct […] then it is sent.”* | *“And, of course, everything has to be listed and displayed in such an app, or what has been ordered, when the delivery might be announced, but still has to be canceled again due to weather conditions, because the drone just can't get to the destination.”* | (All important quotes in the article) |
| Handover Usability | *“As far as the anesthetics or general security is concerned, I think you can do a two-way encryption. Then it's just a box that is electronically locked. And I have to enter a code or whatever.”*  *“So, it flies to a predefined GPS coordinate. And for the fine adjustment it then looks with the camera, where is my QR code. And then lands exactly on it. Theoretically, of course, you could also put that into an app like this. That the first time you get a target QR code, which you print out on A4 and then put somewhere. And then the system knows just exactly where I have to go.”* | *“The requirements for the app would then actually be a documentation that the delivery has actually been made. I think that's very important.”* | (All important quotes in the article) | (All important quotes in the article) |
| Accessibility | (All important quotes in the article) | *“But we are health professionals. We are not a pill distributer. That´s the crucial difference.”* | *“But from a pandemic standpoint, it worked. It had to work. We all pulled together, pharmacy and us. And then it worked.“* | *“[…] how much it will be used.”* |
| Concerns | *“And then it wouldn't be feasible at all. Especially in rural areas, where the logistical connection is difficult, the older people can't handle the technology. And then the drone is not quite operational.”* | They wondered where they include the patient: “*How do I include him in a way that doesn't make the customer feel over-advised or that he then jumps off and says: I don't want to, it's not important to me.”*  *“[...] and if then my anesthetics is in my neighbors garden and the small child takes the medicines. Who is then the one with the insurance?”* | *“And then there's just the question of whether the patient can assess that? For example, how much space does the drone need to land?”*  They were concerned *“[…] that this ping-pong communication that takes place at the counter, that would actually only work one-sided. That could lead to problems.”* Regarding communication problems they also talked about language *“[…] barriers, if the user is not German-speaking now.”*  *“And they've stopped it now after two or three years because it was too complicated.”*  Moreover, nurses questioned *“[…] what happens, for example, if the drone injures someone. Or I'm elderly and can't get out, but outside is my grandson. All of a sudden, the drone arrives. The grandchild doesn't get it that he has to get out of the way now, and the drone hurts the child. What happens then? So that's always the big question now with autonomous driving, when the car does run over someone.”* | *“This is how I have experienced it. The police was standing in front of our door, the health department was standing in front of our door, and the others go to work and think, "Why is the police car here?" You know, I was crying. Maybe it's easier in big cities, because you're running around anonymously. But how is it really when you are now supplied by such a drone and the whole world knows: You caught Corona.”*  *“I experienced that firsthand with my mother, who lives in the country. We were all flat on our backs and my mother couldn't even get up. I didn't know what to do. I phoned around with the health department lying in bed with a fever and no one took care of my mother. But then, fortunately, a general practitioner agreed and went to see her on Sunday. And now she always says: “The general practitioner saved my life”. It wasn't about the medication at all.”*  One patient said: *“That pisses me off, everything is only available via the cell phone. If the cell phone is gone, what then? If you've misplaced it or forgotten it or it's broken, then you don't have anything anymore.”*  *“We have Wi-Fi to some extent, but you can't really make phone calls either. I don't know how it is in rural areas. There are still so many dead spots where nothing works.”* |
