# Supplementary material for: “Of course, drones delivering urgent medicines are necessary. But I would not use them until…” Insights from a qualitative study on users’ needs and requirements regarding the use of medical drones
Source: PLoS One. 2023 May 8;18(5):e0285393. doi: 10.1371/journal.pone.0285393 (PMC10166545; doi:10.1371/journal.pone.0285393)
Supplement: Attachment — Submitted filename: PONE-D-22-13044_reviewer-SAJ.pdf [file pone.0285393.s005.pdf]

PLOS ONE

“Of course, drones delivering urgent medicines are necessary. But I would not use them until…” Insights from a qualitative study on users’ needs and requirements regarding the use of medical drones

--Manuscript Draft--

**Manuscript Number:**	PONE-D-22-13044
**Article Type:**	Research Article
**Full Title:**	“Of course, drones delivering urgent medicines are necessary. But I would not use them until…” Insights from a qualitative study on users’ needs and requirements regarding the use of medical drones
**Short Title:**	User’s requirements of drone-based medication delivery
**Corresponding Author:**	Franziska Stephan<br>Martin-Luther-Universität Halle-Wittenberg: Martin-Luther-Universitat Halle-Wittenberg<br>Halle, GERMANY
**Keywords:**	health services research; medical delivery; drones; user-centred framework; healthcare
**Abstract:**	Background.<br>The current COVID-19 pandemic, demographic trends, and the increasing shortage of skilled workers pose major challenges for the care of people with and without care needs. The potential of drones as unmanned aerial vehicles in health care is being discussed as an effective innovative way of delivering much-needed medicines, especially in rural areas. Although the advantages are well known, the needs of the users have not yet been taken into account.<br>Methods.<br>Online-based focus groups were conducted with actors from different disciplines (nursing, pharmacy, physicians) with a total of n=36 participants. The focus was primarily on potential problems, needs, and requirements of the users regarding the use of drones. Structured and contrastive snowball sampling has been deployed. The focus groups were fully transcribed and analyzed for content [1].<br>Results.<br>Especially during the pandemic situation, delays and restrictions in the delivery of medicines have been noticed. All interview partners (patients, pharmacists, physicians and nurses) see drones as useful in cases of limited mobility, time-critical medicines (rapid availability), emergencies and disasters (e.g., floods), but also for the delivery of regular medicines in rural areas (e.g., for the treatment of chronic diseases).<br>Discussion.<br>Drone deliveries do not play a role in the health system yet despite their great importance, which is perceived as particularly evident in the pandemic situation. The results lead to the conclusion that this is mainly due to knowledge and application deficits, so that educational and advisory work is absolutely necessary. There is also a need for further studies that go beyond the scope of acceptance research to describing and evaluating concrete scenarios of drone delivery on the basis of a user-centred approach.
**Order of Authors:**	Franziska Stephan
	Denny Paulicke
	Martin Grünthal
	Patrick Jahn
**Additional Information:**	
**Question**	**Response**
**Financial Disclosure**<br><br>Enter a financial disclosure statement that	This research was funded by the Federal Ministry of Education and Research Germany<br>through the “TDG - Translational region of Digital Health Care” project [03COV25E]. PJ

Powered by Editorial Manager® and ProduXion Manager® from Aries Systems Corporation

describes the sources of funding for the work included in this submission. Review the submission guidelines for detailed requirements. View published research articles from *PLOS ONE* for specific examples.<br><br>This statement is required for submission and **will appear in the published article** if the submission is accepted. Please make sure it is accurate.<br><br>**Unfunded studies**<br>Enter: *The author(s) received no specific funding for this work.*<br><br>**Funded studies**<br>Enter a statement with the following details:<br>• Initials of the authors who received each award<br>• Grant numbers awarded to each author<br>• The full name of each funder<br>• URL of each funder website<br>• Did the sponsors or funders play any role in the study design, data collection and analysis, decision to publish, or preparation of the manuscript?<br>• **NO** - Include this sentence at the end of your statement: *The funders had no role in study design, data collection and analysis, decision to publish, or preparation of the manuscript.*<br>• **YES** - Specify the role(s) played.<br><br>* typeset	receives this funding. The funders did neither partake in study design, data collection, analysis, nor in the preparation of the manuscript or the decision to publish.
**Competing Interests**<br><br>Use the instructions below to enter a competing interest statement for this submission. On behalf of all authors, disclose any competing interests that could be perceived to bias this work—acknowledging all financial support and any other relevant financial or non-financial competing interests.<br><br>This statement is required for submission and **will appear in the published article** if the submission is accepted. Please make sure it is accurate and that any funding	The authors have declared that no competing interests exist.

sources listed in your Funding Information later in the submission form are also declared in your Financial Disclosure statement.<br><br>View published research articles from *PLOS ONE* for specific examples.<br><br>**NO authors have competing interests**<br><br>Enter: *The authors have declared that no competing interests exist.*<br><br>**Authors with competing interests**<br><br>Enter competing interest details beginning with this statement:<br><br>*I have read the journal's policy and the authors of this manuscript have the following competing interests: [insert competing interests here]*<br><br>* typeset	
**Ethics Statement**<br><br>Enter an ethics statement for this submission. This statement is required if the study involved:<br><br>• Human participants<br>• Human specimens or tissue<br>• Vertebrate animals or cephalopods<br>• Vertebrate embryos or tissues<br>• Field research<br><br>Write "N/A" if the submission does not require an ethics statement.<br><br>General guidance is provided below. Consult the submission guidelines for detailed instructions. **Make sure that all information entered here is included in the Methods section of the manuscript.**	The study was approved by the Ethics Committee of the Martin-Luther-University Halle-Wittenberg (protocol code 2021-069 and date of approval May 6, 2021). All participants gave written informed consent.

**Format for specific study types**<br><br>**Human Subject Research (involving human participants and/or tissue)**<br>• Give the name of the institutional review board or ethics committee that approved the study<br>• Include the approval number and/or a statement indicating approval of this research<br>• Indicate the form of consent obtained (written/oral) or the reason that consent was not obtained (e.g. the data were analyzed anonymously)<br><br>**Animal Research (involving vertebrate animals, embryos or tissues)**<br>• Provide the name of the Institutional Animal Care and Use Committee (IACUC) or other relevant ethics board that reviewed the study protocol, and indicate whether they approved this research or granted a formal waiver of ethical approval<br>• Include an approval number if one was obtained<br>• If the study involved *non-human primates*, add *additional details* about animal welfare and steps taken to ameliorate suffering<br>• If anesthesia, euthanasia, or any kind of animal sacrifice is part of the study, include briefly which substances and/or methods were applied<br><br>**Field Research**<br><br>Include the following details if this study involves the collection of plant, animal, or other materials from a natural setting:<br>• Field permit number<br>• Name of the institution or relevant body that granted permission	
**Data Availability**<br><br>Authors are required to make all data underlying the findings described fully available, without restriction, and from the time of publication. PLOS allows rare exceptions to address legal and ethical concerns. See the PLOS Data Policy and FAQ for detailed information.	Yes - all data are fully available without restriction

A Data Availability Statement describing where the data can be found is required at submission. Your answers to this question constitute the Data Availability Statement and **will be published in the article**, if accepted.<br><br>**Important:** Stating 'data available on request from the author' is not sufficient. If your data are only available upon request, select 'No' for the first question and explain your exceptional situation in the text box.<br><br>Do the authors confirm that all data underlying the findings described in their manuscript are fully available without restriction?	
**Describe where the data may be found in full sentences. If you are copying our sample text, replace any instances of XXX with the appropriate details.**<br><br>• If the data are **held or will be held in a public repository**, include URLs, accession numbers or DOIs. If this information will only be available after acceptance, indicate this by ticking the box below. For example: *All XXX files are available from the XXX database (accession number(s) XXX, XXX.).*<br>• If the data are all contained **within the manuscript and/or Supporting Information files**, enter the following: *All relevant data are within the manuscript and its Supporting Information files.*<br>• If neither of these applies but you are able to provide **details of access elsewhere**, with or without limitations, please do so. For example:<br><br>*Data cannot be shared publicly because of [XXX]. Data are available from the XXX Institutional Data Access / Ethics Committee (contact via XXX) for researchers who meet the criteria for access to confidential data.*<br><br>*The data underlying the results presented in the study are available from (include the name of the third party*	All relevant data are within the manuscript and its Supporting Information files.

and contact information or URL).<br>• This text is appropriate if the data are owned by a third party and authors do not have permission to share the data.<br><br>* typeset	
Additional data availability information:	

Powered by Editorial Manager® and ProduXion Manager® from Aries Systems Corporation

Manuscript
Click here to access/download;Manuscript;ADApp_focus group_manuscript_PLOS ONE.docx

Running Head: User's requirements of drone-based medication delivery

"Of course, drones delivering urgent medicines are necessary. But I would not use them until…" Insights from a qualitative study on users' needs and requirements regarding the use of medical drones

Franziska Stephan[1,2*†], Denny Paulicke[1,3†], Martin Grünthal[4], Patrick Jahn[1,2,]

[1] Health Service Research Working Group | Acute Care, Department of Internal Medicine, Faculty of Medicine, University Medicine Halle (Saale), Martin-Luther-University Halle-Wittenberg, Halle (Saale), Germany

[2] Faculty of Medicine, Martin-Luther-University Halle-Wittenberg, Halle (Saale), Translationsregion für digitalisierte Gesundheitsversorgung (TDG), Halle (Saale), Germany

[3] Akkon University of Human Sciences, Department of Medical Pedagogy, Berlin, Germany

[4] Pharmacy at the Bauhaus, Dessau-Roßlau, Germany

* Corresponding author

E-Mail: franziska.stephan@uk-halle.de

[†]Both authors contributed equally.

Abstract

Background.

The current COVID-19 pandemic, demographic trends, and the increasing shortage of skilled workers pose major challenges for the care of people with and without care needs. The potential of drones as unmanned aerial vehicles in health care is being discussed as an effective innovative way of delivering much-needed medicines, especially in rural areas. Although the advantages are well known, the needs of the users have not yet been taken into account.

Methods.

Online-based focus groups were conducted with actors from different disciplines (nursing, pharmacy, physicians) with a total of n=36 participants. The focus was primarily on potential problems, needs, and requirements of the users regarding the use of drones. Structured and contrastive snowball sampling has been deployed. The focus groups were fully transcribed and analyzed for content [1].

Results.

Especially during the pandemic situation, delays and restrictions in the delivery of medicines have been noticed. All interview partners (patients, pharmacists, physicians and nurses) see drones as useful in cases of limited mobility, time-critical medicines (rapid availability), emergencies and disasters (e.g., floods), but also for the delivery of regular medicines in rural areas (e.g., for the treatment of chronic diseases).

Discussion.

Drone deliveries do not play a role in the health system yet despite their great importance, which is perceived as particularly evident in the pandemic situation. The results lead to the conclusion that this is mainly due to knowledge and application deficits, so that educational and advisory work is absolutely necessary. There is also a need for further studies that go beyond the scope

of acceptance research to describing and evaluating concrete scenarios of drone delivery on the basis of a user-centred approach.

Introduction

The COVID-19 pandemic poses major challenges to the provision of care for the elderly and those in need of long-term care. Particularly in rural regions with a decreased level of infrastructure, where care providers have to overcome large geographical distances, it becomes evident that new, innovative solutions are needed to cope with the pandemic situation.

General social trends (e.g., increasing urbanization/rural exodus, etc.) combined with supply bottlenecks in rural regions have made it increasingly difficult to provide health care to the people living there. The infection control measures taken in the context of the COVID-19 crisis have exacerbated this effect and made it visible, since the elderly and people in need of care in this special situation are unable to provide themselves with the urgently needed medicines [2,3].

The use of digital technologies in health care is also increasingly being discussed for this reason. Amid the ongoing COVID-19 pandemic, the Internet of Medical Things (IoMT) becomes more and more a focus in health care, which reduces the burden on health care systems. IoMT is an amalgamation of software applications and medical devices that are connected to health care IT systems for offering an extensive health care service. This includes monitoring, medication tracking, and wearables for transmitting health information [4]. In this regard, one possibility is to overcome large distances with the help of drones and thus reach people on the ground. In general, drones are widely used in medicine and health care in the areas of public health and disaster relief, telemedicine, and medical transport [5]. In public health, drones have been used to collect information on the number of patients in need, identify health threats and for epidemiological research. In telemedicine, drones have been used for tele-surgery and remote diagnosis. Drones in medical transport have been widely used for the delivery of medicines (e.g., vaccines, drugs), blood supplies, organs, defibrillators, and other medical supplies from one medical facility to another or to (COVID-19) patients [4,6]. In a recent review, Hiebert

et al. [7] identified the delivery of medical supplies and treatments as the most common application for drones in health care. Medical drones have an immense benefit to health care. They improve response times of emergency services, access to health services in difficult and remote areas, and clinical outcomes (e.g., survival after cardiac attack) [7]. Moreover, medical drones are considered a more cost-effective alternative for air transport of medical supplies compared to helicopters in mountainous, desert, and forest areas where there is no access to roads or long distances, or in areas that have been affected by major natural disasters [8]. Although, the potential of drones in health care is considered high, technological, organizational, and environmental factors (TOE barriers) affect the use and performance of drones [7,9]. Due to structural/organizational conditions and processes that have not yet been defined (legal conditions, framework conditions, etc.), the use of drones in Germany and many other countries is still limited to research projects and prototypes [7,10]. The majority of studies were located in Africa due to more regulatory openness compared to Europe and Northern America [10,11]. Nevertheless, studies conducted in Africa cannot simply be transferred to European contexts [12]. However, the most identified factor that affects drone use was the usability and design of drones [7]. In this vein, user acceptance issues lack of user's skills and expertise, letting negative perception and technology immaturity hinder the use of drones [9]. A positive attitude toward drones affect the willingness to use and interact with them for different purposes. Moreover, the technical interest in general and knowledge about drones are identified as supportive factors for acceptance [13]. According to the technology acceptance model (TAM), the attitude toward using a technology is dependent on perceived ease of use and usefulness. In other words, better usability and higher usefulness of a technology lead to a higher acceptance of the same [14,15]. One way to further increase the acceptance of drones could be the engagement of users in designing and developmental processes to consider user's needs and requirements within the user drone interaction. Even though, particularly in the COVID-19 pandemic, awareness of new and innovative forms of care has grown further [4], the centering users for medicine delivery with

drones is little the focus of research. In a recent review, it was found that there are currently no studies that examine the interaction between humans and drones in the delivery of medicines [12]. The study also shows that there are hardly any approaches to explaining the needs and views of potential users of drones.

To further our knowledge of the potential for drone delivery of medicines, a feasibility study is underway in the region of Saxony-Anhalt, Germany, known as ADApp-project (German: Apotheken-Drohnen-App). The concept is to combine a pharmacy app with drone logistics for the delivery of urgent medicines or contactless delivery for comprehensive medication supply, mainly in rural areas. Against the backdrop of the current pandemic situation, scenarios for alternative solutions for the logistics of medications are being developed in a co-creative process together with users and developers, which can be taken over by the drone quickly and without contact, e.g., in the case of acute medication requirements in respiratory emergencies. The ADApp is a novelty for the German region mentioned above. Currently, drones are already being tested for health care logistics between clinics in large cities (e.g., Munich [16]) or used for health care in rural areas outside of Europe (e.g., in Africa [17–20]).

Special about ADApp is that, for the first time, the entire supply process can be coordinated digitally, from the issuing of the e-prescription at the general practitioner, to the order at the pharmacy, to delivery by drone directly to the patient's doorstep or to an institution. We consider after the current common proof of concepts flights the legally, administrative and logistically integration of drones within the continuous supply process as the next important step to reach practical implementation. For this reason, the feasibility study is using a phased user-centered design to explore the needs and requirements of users as well as the practical and logistical facilitators and challenges involved in the development and implementation of such technology. The aim of the project is to design an app and drone system in a way that increases usability, acceptability, and satisfaction of basic psychological needs in order to evaluate the effectiveness of using app-assisted drone-based medicine delivery. Users' involvement at every

developmental step will ensure the feasibility of medical drone delivery (see **Figure 1**). A monocentric exploratory mixed-method study design with multiple measurement points has been chosen. As one part of the feasibility study, a qualitative study has been conducted in order to identify ideas, needs, and requirements of all interacting users of a drone-based medicine delivery. This paper explores the question of what user requirements are in the delivery of medicines with drones under current pandemic conditions and beyond. The focus is on the digital process of a pharmacy drone app and the human-drone interaction. The aim is to identify the requirements of the users and to show which conditions must prevail for implementation under pandemic conditions.

Methods

ADApp Design

The present study design is embedded in a broader context of user-centred framework oriented at Farao's and colleagues modified *Information System Research framework incorporating modes of design thinking* [21]. The classical *Information System Research framework (ISR)* uses three research cycles, where the environment of users is understood (relevance cycle), objects relating to the problem are created (design cycle), and findings from the evaluation are combined with the existing knowledge base (rigor cycle) [22]. An example that has been used in ISR is the technology acceptance model (TAM; [14,15]), which shows that better usability and higher usefulness of a technology lead to a higher acceptance of the same. The *Design Thinking (DT)* approach is used to prioritize the needs of users to solve complex problems [23,24]. It includes different steps. It starts with the identification of needs with the initial understanding of the users and the problem itself (*empathize*). The user feedback is afterwards

analyzed into identifiable needs (*define*). The next mode is the *ideation*, which involves generating diverse ideas that in the next step are translated from thoughts to physical representations (*prototyping*). The next mode is *testing*. Its purpose is to refine and improve prototypes as well as simulating the use in the context. In a final step (*implementing*), the results are evaluated in medium-term and long-term change which can result in the first step, the identification of needs (Altman, 2018). Taken together, ISR starts from typical, observed problems in practice and solves them with scientific principles at a high level of abstraction. DT identifies additional aspects of the design process by focusing on the needs and requirements of users [25]. Thus, Farao et al. [21] combined these two approaches, which can be used for desigining and re-designing technologies. This approach (1) involves the environment (ISR: relevance cycle) as well as problems, needs, and requirements of users and companies (DT: empathize and define mode); (2) promotes the transferability of the design knowledge (ISR: rigor cycle) through scientific theories and models (e.g., the technology acceptance model), results of existing artefacts (e.g., literature reviews), and through testing (DT), which includes the engagement of users (e.g., evaluation of functionalities); (3) centers the users throughout the design process by users involvement during technical development (ISR: design cycle, DT: ideation and prototyping), which in fact contributes to the knowledge base and involves the needs of users at all times (see **Figure 1**).

Figure 1. A. Users' involvement at every developmental process.

Users' needs are scientifically assessed within the relevance cycle and evaluated within the rigor cycle which contributes to the knowledge base. The knowledge base contributes to the adaptation of technical development based on the translation of scientific findings into technical ideas. Users' needs are incorporated into technical development and technical development is adapted to users and the knowledge base within the design

cycle. Technical development is tested and evaluated with users within the relevance cycle.

B. The whole ADApp study design.

Based on the cyclic and iterative nature of the user centered approach, at all stages the users' needs, technical development, and knowledge base incorporate all conditions of the cycles seen in figure 1 A. Nevertheless, the study started with a literature review about the human drone interaction in the delivery of medical supplies. Scientific theories, such as the METUX model (Peters et al. 2018) or TAM model [14,15], contribute to the knowledge base which were important for choosing the study design. Next, focus groups (FG) were conducted that aimed at collecting the problems, needs, and requirements users have if they imagine using such technology (the present study). Four user groups were defined: general practitioners, pharmacists, nurses, and SAR-CoV2-infected patients. In this step, it was essential that the user's needs are translated into a set of functional requirements and design guidelines. Thus, potential themes were identified at the basis of transcriptions and coding of text segments of FG. These themes are relevant to the software system goals. Thus, prototypes can be sketched for eliciting feedback form users. This is essential for a deeper understanding of the intended goal of an app or technology. A core group will be established, consisting partly of participants of the FG. With the help of walkthroughs and usability testing, the technology will be evaluated and refined iteratively through three evaluation circles. Walkthroughs will be conducted in that participants are encouraged to think aloud and provide feedback on the difficulties and facilitators during the experiencing of the app and drone. It aims at ensuring that the performance of the app and drone matches the user requirements. These walkthroughs will be analyzed qualitatively. For assessing the usability, acceptability, and the satisfaction of psychological needs, the users answer a set of questionnaires

(TUI, SUS, METUX-questionnaires). These questionnaires will be analyzed quantitatively. At this step, three different adhoc groups will be established at each walkthrough which act as control groups. We consider this controlled approach as important to deal with potential biasing effect due to response shift through multiple feedback cycles [26]. In a last step, participants will engage with the app and drone in a natural environment (airport) by aiming at assessing how the participants interact with the technology. Usability and acceptability will be assessed qualitatively with discussions and quantitatively with questionnaires [27]. METUX: Motivation, Engagement, Thriving in User Experience; TAM: Technology Acceptance Model; TUI: Technology Usage Inventory; SUS: Usability Scale Assessments.

Study Setting and Sampling

The present study represents a section of the whole ADApp design by investigating users' problems, needs, and requirements within the relevance cycle by conducting semi-structured discussions (focus groups; FG) which were evaluated within the rigor cycle by using content analysis. The ADApp study was approved by the Ethics Committee of the Martin-Luther-University Halle-Wittenberg (protocol code 2021–069 and date of approval May 6, 2021). All participants gave written informed consent. Results of FG substantiate the further empirical procedure within the framework of the overall project. The discussion thereby pursued the goal of eliciting the subjective relevance frames of the participants in relation to the app and drone use to be developed. For improving the rigor, comprehensiveness, and credibility of reporting methods and results of the present FG study, we followed the COREQ Checklist [28]. The checklist is provided in the additional file S1 Table.

Participants

Between June 2021 and October 2021, we collected data from 36 participants in total. Participants were 44.4% female and 55.6% male, ranging in age from 23 to 75 years (mean 50.1 ± 13.8). We selected participants representing the potential users of the drone based medication delivery service, of the supply chain, and its technical components. Thus, participants were selected according to the role characteristics: general practitioners, nurses, pharmacists, and SARS-Cov2 infected-patients with rehabilitative treatment due to Long COVID condition resulting in four different FG. The FG were conducted separately and uniquely for each role characteristics resulting in four FG: general practitioners (*n* = 8), pharmacists (*n* = 8), nurses (*n* = 6), and COVID-19-patients (*n* = 14). Participants were recruited with the support of the ADApp project team, the Paracelsus Harz-Clinics Bad Suderode, the Medical Journal (Deutsches Ärzteblatt), the Pharmacy-ADHOC Journal (Apotheke-Adhoc), and the DBfK - The German Nurses Association (Deutscher Berufsverband für Pflegeberufe DBfK e.V.). Thus, the selection was purposive and followed the snowball-sampling. Participants were approached by telephone or via email. There was no relationship to participants prior to study commencement. All participants were informed about the procedure, the general aim, and reasons of the study and gave written informed consent before starting the discussion.

Materials

The FG instrument was developed through a multi-stage systematic process and was critically reflected in the context of a quality-assuring feedback and several iteration loops (peer group: project partners) [29]. The FG instrument consisted of seven main types of questions. These questions were based on the Technology Usage Inventory (TUI) [15,30]. The TUI covers four technology-specific and psychological factors assessing the actual use of technology for explaining technology acceptance. Three technology-specific factors and four psychological factors of the TUI were used for question development: usability, usefulness, and accessibility

as well as curiosity, interest, skepticism, and fearfulness. The first, introductory questions, explored the problems of getting care and supply under pandemic conditions and the knowledge and competence in the handling with medication apps and drones (category: problems, knowledge, and competence; TUI factor: curiosity and interest). One key question provided information about how useful the participants find a drone-based delivery of medicines (e.g., “Would you use the pharmacy drone app? Why or why not?”; category and TUI factor: usefulness). Three further key questions asked about participants’ needs in the personal contact in the process of drone delivered medicines (communication), the perception of the process itself (e.g., “How would the process be as barrier-free as possible?”), and the handover (e.g., “What can the physical transfer of the drug look like from your perspective?”). These questions were assigned the factor of usability and specified according to the usability of communication, process, and handover. Another question was about the accessibility of the application and drone delivery (e.g., “What information channels do you use to learn about such technologies?”; category and TUI factor: accessibility). Closing questions provided space for additional thoughts and statements as well as skepticism and fearfulness of using such technology (e.g., “Do you have any other suggestions or concerns you want to let us know?”; category: concerns; TUI factor: skepticism and fearfulness).

Data Collection

The FG were conducted online via *WebEx* with exception of the patient group which was conducted during their rehabilitation stay at the Paracelsus Harz-Clinic Bad Suderode. During the FG discussion, two moderators (M1 and M2) guided the groups. Both moderators have introduced themselves by reporting their credentials and position in the ADApp project. M1 (first author: FS; PhD; research associate; female) controlled the communication process, provided the questions or prompts. The aim was to encourage the participants to discuss and to generate a creative atmosphere by taking up different ideas and opinions so that the participants

can build up narrative ambitions through positive feedbacks [31,32]. M2 (first author: DP; PhD; research associate; male or last author: PJ; PhD; Head of Working Group; male) simultaneously collected thoughts and aspects on an online flip chart (*Miro Board*) for orientation. These were taken up again later in the discussion. During the open narrative phases of the participants, M2 noted additional information of the interactions or the atmosphere of the discussion in order to obtain a later assignment to the data to be evaluated. It serves the purpose of exploration [33]. All moderators provide a high level of expertise and experience in qualitative methods whereby DP proves the most experience in conducting focus groups. Depending on the situational assessment, FG lasted between 60 and 120 minutes [32]. During FG discussion no one else was present besides the participants and the two moderators. At the end of the discussion, the participants had the possibility to leave further contact data in order to encourage participants for further participation and continuous monitoring ("core group").

Data Analyses

The notes taken by M2 were made available to participants and were discussed in the FG, so they could verify that their statements were noted without distortion. However, transcripts were not returned to participants. The participants' responses were audio recorded, transcribed by the company "abtipper", and coded with the help of the program "f4analyse 2". Due to the primary content-related objectives of the research interest and the research question, a simple transcription system was used [34,35]. Thus, only the manifest content of the transcripts were analyzed [1]. The data analysis was carried out according to the content analysis method from Elo and Kyngäs [1]. After becoming familiar with the transcripts by reading them several times, analyses were conducted using a deductive approach moving from the general to the specific. The unit of analysis was defined as words, one or several sentences that contain one theme and was coded in terms of a structured analysis matrix, i.e., only aspects that would fit the matrix were chosen from the data. The categories were prepared in tabular form, so that an

independent evaluation was possible. FS coded transcripts for the categories. DP coded 25% of the transcripts independently and peer examination was conducted by the Health Service Research Working Group and Acute Care with extensive experience in qualitative research.

The data coding followed the categories underlying the FG instrument based on the TUI assessment [15,30]: problems (i.e., curiosity and interest), usefulness, usability (split into communication, process, and handover), accessibility, and concerns (i.e., skepticism and fearfulness) (see **Table 1**). However, these categories were divided into different issues aiming at translating the main categories into a set of functional requirements and design guidelines relevant to the software system goals (see result section). The knowledge base has been queried with a short question of whether the participants have experience and knowledge about drones and medication apps.

Table 1. Structured analysis matrix. The category of usability was subdivided into communication, process, and handover.

No.	category	definition	example
1	***problems***	describes current problems that justify the usefulness of the technology	"At the moment, we have a lot of isolated solutions that make us more work overall."
2	***usefulness***	describes the advantages, the benefits of the technology	"So I think palliative care is of course an important point, for us and for the rural area and then of course elderly patients who need their basic medication."
3	***usability: general***	describes the necessities/ conditions for the comprehensibility, application, and usability of the technology	“I would only use it if it is somehow even remotely compatible with my software.”
3.1	*usability: communication*	describes the necessities/ conditions for the communication (e.g., chat, video, call, etc.) of the technology	"But I think a quick answer is also important. Especially when you're now in quarantine, a bit on your own and maybe scared. If you need a medicine, you're usually not well and I think it's very important that you get an answer as quickly as possible."
3.2	*usability: process*	describes the necessities/ conditions/characteristics of the process of technology	"There must be one drone launch per patient at the end of the day."

3.3	*usability: handover*	describes the necessities/ conditions for the transfer of medication	“One of the requirements for the app would be a documentation that the delivery has actually been made. I think that's very important."
4	***accessibility***	describes the way of access to the technology, the related effort and the necessary resources (technical, economical, financial, organizational)	"The prerequisite would then actually also be a wireless connection for the app."
5	***concerns***	describes the dangers, the risk, the complications, the disadvantages, the difficulties that the technology brings with it	"So I think if someone has small children or dogs it's kind of difficult."

Results

Participant’s Knowledge Base

According to the knowledge and competence in the handling with drones, only five participants out of 30 (16.7%) stated that they already have experiences with drones. General practitioners are having the most experience (100% out of three responses). Ten out of 31 participants (32.2%) were familiar with the handling with medication apps, whereas pharmacists (85.7% out of seven responses) and general practitioners (75% out of four responses) are having the most experience. However, the patients indicated no competences in handling medication apps. Further results are reported in **Table 2**.

Table 2. Participant characteristics (*n* = 36).

	**general practitioners**	**pharmacists**	**nurses**	**patients**	**total**
n	8	8	6	14	36 (30)
age	47.5 ± 10.0	44.14 ± 9.6	39.5 ± 13.4	57.8 ± 14.2	50.1 ± 13.8
age range	30 - 57	34 - 61	26 - 58	23 - 75	23 - 75
women (%)	3(37.5)	2(25)	3(50)	8(57.1)	16(44.4)

men (%)	5(62.5)	6(75)	3(50)	6(42.8)	20(55.6)
drone experience (%)	3(100) *n* = 3	1(14.3) *n* = 7	0 *n* = 6	1(7.1) *n* = 14	5(16.7) *n* = 30
medication app experience (%)	3(75) *n* = 4	6 (85.7) *n* = 7	1(16.7) *n* = 6	0 *n* = 14	10(32.2) *n* = 31

Qualitative content analysis results

Different issues were extracted for five main categories. We identified five different problems (category 1), eleven issues of usefulness (category 2), seven issues of general usability (category 3), five of communication usability (category 3.1), six of process usability (category 3.2) as well as three of handover usability (category 3.3), ten issues of accessibility (category 4), and twenty different concerns (category 5). All issues were assigned to one of three factors, classified into technological, organizational, and economical (TOE) factors, influencing adoption of drones [9] (see **Table 3**). **Figure 2** shows the proportion of five main categories in relation to total utterances illustrated in percentage. It shows that the categories of usability (usability total) and concerns accounted for the majority of the discussions. General practitioners discussed most about within the usefulness category of drone-based medication delivery while pharmacists debated most about topics that match usability categories. Within the usability subcategories, the handover was the most debated usability issue. Nurses conversed most about possible concerns and difficulties involved in the drone delivery. However, the concerns had the most discussion compared to the other categories.

Figure 2. Proportion of categories.

Proportion is per total utterances for pharmacists, general practitioners, nurses, and COVID-19-patients for the categories: problems, usefulness, usability, accessibility,

and concerns. Usability is subdivided into general, communication, process, and hand-over. All usability subcategories are merged into usability total.

Problems

COVID-19-patients experienced no support during their disease or during the pandemic situation, especially in rural areas. *"But when you've had Covid, I layed for four weeks, the 5th week they did a little gymnastics with me. When I came home, I could barely walk 5 meters, that's how tired I was. I only gradually reacquired that with my own training. Nobody supports you, no hospital, especially if you live in a rural village."* However, pharmacists stated that a drone for COVID-19 aftercare would have been useful. Furthermore, patients and pharmacists commented that the supply in rural areas is limited. This is mainly due to the fact that young people move to the city and the older ones stay in the village. Some can no longer drive a car and are therefore dependent on the care of others which is not only an issue during pandemic situations: *"There are 30 people living in our village. Out of the 30 people, 12 are over 80, then we have about 13 people who are over 60, and then we actually have only a handful of young people. Almost everyone has an own house, the children have moved out. For them the idea is very good with drone delivery, that's really nice I must say. The oldest in our village is 95, he can't drive a car anymore, and he always has to ransom someone from the family to do it."* (patients). The pandemic situation is not only having an impact on patients. General practitioners and nurses are reporting restrictions to office hours that result in a decrease in treatments which in turn leads to delayed diagnoses. Moreover, the general practitioners said that it was difficult for them to stay in contact with the patients without physical contact which was prone to errors. They *"[...] gave the prescription by mail. And if there had been a mistake, the patient would have had to report it. But we couldn't recheck that now."* They also reported delay in medication delivery due to the pandemic situation. A nurse said: *"Certain medicines were simply missing in the morning."* The nurses also stated out that *"[...] due to the pandemic, we*

had patients at the beginning who said we didn't want any visitors because they were afraid that we would bring Corona." General practitioners noted that independently of the pandemic situation, rush hours in cities caused delays in medication delivery, too: *"That's where our couriers take so long to get from Berlin-Mitte to Berlin-Wedding at rush hour."* Pharmacists emphasized the increasing palliative care, where rapid delivery is necessary. Nevertheless, they reported *"[...] we have a lot of isolated solutions that are making more work overall."* Additionally, they talked about the lack of economic efficiency of digital solutions which however could facilitate their work.

Usefulness

Fitting to the problems of rural care and the lack of support during Covid-19, all sample groups agreed that a drone-based medication delivery is useful in cases of immobility or limited mobility. They mentioned for example the supply of rural areas or areas with poor (pharmacy) infrastructure, long distances, and heavy traffic or single/fewer mobile people who have no connection to care by the outpatient care service, e.g. in case of quarantine or after surgeries. General practitioners and nurses strengthened this by a rapid availability and delivery of time-critical medicines to patients at home or to clinics and outpatient care service. However, patients and general practitioners also noted the importance of the delivery aspect. They find it useful in cases of emergency (e.g., pandemic situations) and disaster (e.g., flooding) and emphasized the advantage of contactless delivery. In addition, they felt that it would be important for drone-based medication delivery to be demand-driven. They suggested to include the delivery of regular medicines to patients and institutions (e.g., in the care of chronic diseases).

Independent of the pandemic or emergency situations, the participants indicated the usefulness on the relief for pharmacies, patients, and general practitioners (patients), the support for local pharmacists (patients) through an increase in attractiveness (pharmacists), the support for environment (e.g., fewer cars (patients)), the reduction of medication retention in clinics

(general practitioners), the delivery of medical and laboratory products or blood (general practitioners), and the reduction of logistical processes (general practitioners). Pharmacists mentioned that the service allows them to be more flexible and innovative. This innovative character was expanded by the idea of *“[...] delivery at night. Because I was often called: “Can't you bring it over to me? I'm a 90-year-old granny. I can't get out of there.” That's why I would use it at night."*

General Usability

This category includes the subcategories “communication,” “process,” and “handover”. However, seven issues are identified as usability that cannot be assigned to the subcategories. In situations where people are in need, immobile, or have less digital competences, patients and nurses proposed speech recognition possibilities. Thus, the ordering medications and the announcement according to the delivery process could be facilitated. Furthermore, patients suggested an additional device so that not everything is smartphone-based which would accommodate people with low digital competences. *"If I'm in a situation where I'm in need of help, then such an additional device can really be a blessing. I think everyone has to weigh it up for themselves. I could imagine that a message comes through such a device: “the medication is on the way or that is then there and that is deposited there, in front of the front door, on the terrace.”."*

When talking about usability, issues outside the pandemic and emergency scenario were also raised. Patients and nurses reiterated the emphasis on need-based delivery that delivery should also be extended to regular medications (e.g., chronic diseases). Especially nurses said that the medications should be delivered to the outpatient care office. Thus, the app should indicate when the outpatient care service has office hours during which the medication can be delivered, and general practitioners should be able to see in the app which nursing service is responsible for a certain patient. *"I imagine if the emergency general practitioner writes a prescription at night, he can see that nursing service XY is responsible for this patient, and would*

then perhaps send the prescription to the responsible nursing service so that the nursing service can bring the medication in the morning." General practitioners and pharmacists talked about the interface integration, i.e., they only would use it if the app was integrated into practice software, web-based with password access or QR code (general practitioners), or provided an interface integration with pharmacy merchandise management systems (pharmacists). The pharmacists emphasized that they would not tolerate a stand-alone solution outside pharmacy technology otherwise it would be more work and therefore unattractive for pharmacists: "*So I would only use it if it is somehow even remotely compatible with my software."* They also indicated that there should be only digital and automated solutions: *"But when we talk about a project like this, it actually has to be completely digital. We can't have any analog steps in between."* Another pharmacists said: *"When the e-prescription is forwarded to the pharmacy, that is fed in right away and the system knows: in stock, can be delivered immediately and is translated into the time I can tell the patient when to expect the delivery."*

For patients it is important that the user has the data sovereignty and is able to delete contact data themselves while general practitioners talked about app security, i.e., that the app should be without errors. Moreover, general practitioners like to have control over the process and the medication. That means, the general practitioners would like to release the medication for the drone-based delivery depending on whether a patient is able to receive it by drone. Pharmacists prefer to have an own drone for more plannability. Furthermore, pharmacists and general practitioners discussed about different drones for different use cases. They suggested scalable drones depending on range, flight speed, and payload (i.e., small drones for urban and rural areas for short distances and individual customers as well as large drones for urban and rural arears for institutions and clinics).

Communication Usability. All participants thought the app needed to have consultation options between general practitioners and patients as well as between pharmacists and patients,

e.g., via video call, chat function, call option, or feedback form, especially when a person is in need or quarantine. *"But I think a quick answer is also important, especially when you are now in quarantine, a bit on your own and perhaps afraid. When you need a medicine, you're usually not well, and I think it's very important that you get an answer as quickly as possible."* (patients). In this vein, pharmacists confirm that perspective: *“Counseling comes first and foremost. We are health care professionals. We want the patient to come first, not our economic interests. [...] similar to tele-pharmacy."* However, not all participants of the general practitioner’s group like to have immediate communication: *“And just have, if necessary, ways of asking back, what it looks like. For example, “I got the medication” - thumbs up. “I know how to use it” - thumbs up. “I tolerate it quite well” - thumbs up. Or just the possibility to say: “I got it, but I am not quite sure how to take it now. Please, give me an answer back or call me or I'll call you right now.”."* Additionally, nurses find it important that they themselves and patients get information about the medication plan (i.e., instructions of what is important with other medications) and medication intake in form of digital instruction leaflets: *"I can imagine that the app might also provide instructions on how to take the medication. I don't know if everyone in the outpatient care service is always familiar with all the medications, how they should perhaps be taken, and instructions perhaps also for the patients themselves.”* Moreover, they also would like to have note functions in the app for delivery notes.

Independent of pandemic situations, patients prefer that drones would be labeled as medical drone for increasing the social acceptance when a drone flies over another property. General practitioners prefer to have partner pharmacies for callback and coordination opportunities: *"Of course, you have to coordinate with the pharmacies beforehand to find out which ones will participate. Because the system only works if I have at least one partner pharmacy that converts the e-prescription into a real medication in the physical world.”*

Process Usability. All sample groups expressed a general necessity of shipment tracking. They would like to have a fixed and transparent process, i.e., information about the process status, fixed contact persons, and fixed delivery times. *"Then I have a hook - e-prescription issued; e-prescription received by pharmacist; drone packed. And the patient acknowledges receipt afterwards and says: "got it."."* (general practitioners). *"And, of course, everything has to be listed and displayed in such an app, or what has been ordered, when the delivery might be announced, but still has to be canceled again due to weather conditions, because the drone just can't get to the destination."* (nurses). Nevertheless, patients noted that they want information limited to a minimum: the request is in process and the drone is arriving at a certain time. They also stated the wish of forwarding the e-prescription from general practitioner directly to pharmacy making the process as lean as possible. The app should display the delivery locations when living in a city and should navigate to the next drop-off location (nurses): *"That the app maybe displays where the next delivery locations are now."* Nurses also like to have an indication of the last deliveries in an order summary. They also find it useful to include the possibility to send the medications to the neighbor. For patients and pharmacists it is important that the operations and processes are as simple as possible. *"When I'm really sick as a patient I should be able to use it. That it is extremely easy to handle purely in terms of function."* (patients).

Bilateralism represents another issue of process usability which was addressed by general practitioners and nurses. Bilateralism means that either the general practitioner issues an e-prescription, or the patient requests an e-prescription (for example in the case of chronic diseases), i.e., the possibility to request a medication and e-prescription from the general practitioner because a medication is needed, or the supply is running low. *"Maybe it goes there, in reality, that you can also request something. That you can trigger that as a nursing service or say, here the supply is exhausted, and that then the corresponding general practitioner writes the prescription."* Nurses also discussed about a photo function in the app, so that the nurse who *"[...] comes to the patient, who just has a visit from the general practitioner now and has a*

prescription lying there […]" can *"[...] scan it and sent it. Maybe also, so for the next day, with us for delivery, or, if it is urgent, for the evening additionally."* However, pharmacists have completely different issues for process usability. They talked about the issue of infrastructure. Especially pharmacists indicated that it is necessary that the app is adaptable to the daily pharmacy routine: *"We always have a pharmaceutical staff checking it. And then it's delivered by a non-pharmaceutical staff. And we can do it the same way with the drone. […] The pharmaceutical staff looks over it again. If all the information is correct [...] then it is sent"."* They expressed the need of drone loading areas that can be reached from more than one pharmacy and the need of physical conditions in pharmacy, e.g., charging possibilities. They also discussed about the availability of the drones. For them it is a requirement that the availability and charging status of the drone is visible, e.g., within the app, so that it is clear whether the drone is ready for use or not.

Handover Usability. The issue of handover usability most discussed by all FG was the identification during medication receipt. *"The requirements for the app would then actually be a documentation that the delivery has actually been made. I think that's very important."* (pharmacists). The participants discussed about different possibilities of identification, e.g., through fingerprint, voice recognition, code entry, electronic signature, images, token exchange, PIN, TAN, or QR code. *"As far as the anesthetics or general security is concerned, I think you can do a two-way encryption. Then it's just a box that is electronically locked. And I have to enter a code or whatever."* (general practitioners). Even if general practitioners know about a certain residual risk because they "[…] *never know one hundred percent what will happen to the anesthetics prescription if* [they] *give it out somehow",* they commented that *"[...] it should be technically controllable"* and possible. Another issue discussed was the form of handover via winch (patients, nurses, general practitioners) or landing (patients, pharmacists). The winch was indicated as mainly useful in urban areas (patients) while landing seems to be a useful scenario

in rural areas (patients, pharmacists) or at institutions with a central landing area (patients, nurses, general practitioners). The general practitioners came up with the idea of *"[...] placing a kind of target QR code somewhere and the drone recognizes with the camera that there is a QR code - there is "my landing location". So it flies to a predefined GPS coordinate. And for the fine adjustment it then looks with the camera, where is my QR code. And then lands exactly on it. Theoretically, of course, you could also put that into an app like this. That the first time you get a target QR code, which you print out on A4 and then put somewhere. And then the system knows just exactly where I have to go."* The pharmacists came up with the idea to deliver the medicine in rural areas to a local central point, e.g., the local lottery store. For urban areas, nurses generated a few ideas for the landing scenario: landing on the roof, landing at a landing pad that can be opened from the outside of buildings or gripper arms at the drone which passes the medication through the window. Patients imagined that the drone could hover and release the medicine with a winch in front of the window so that the patient only needs to open the window and take the medication. Nevertheless, in case of landing, the location must be accessible to the patients or relatives (nurses). However, landing or winch are both scenarios that were accepted by all participants in general. A smaller issue that was discussed was the method of payment. Only patients talked about that issue and said that they would like to pay within the app or by invoice.

Taken together, the main issues discussed by all participants were consulting (communication usability), shipment tracking (process usability), and identification during handover (handover usability). **Figure 3** shows the proportion of three main usability issues per total utterances. It becomes visible that shipment tracking was taken up a lot of discussion space within the patient and nurses sample groups. While the identification during handover was less discussed for patients, it was the biggest issue for pharmacists. Moreover, another big issue a lot discussed by pharmacists and also general practitioners was the counseling.

Figure 3. The proportion of issues of usability categories per total utterances.

Accessibility

In response to the question what information channels they would use to learn about such technologies, the participants talked about four different issues: the access to the technology and the necessary resources related to environment, organization, and technique. With regard to the access to technology, pharmacists want to inform patients and nurses what has also been reflected by patients and nurses. Patients and nurses also would like to have information about broad media such as newspaper, television, social media, or flyer. Moreover, patients suggested cooperation with health insurance companies so that the information about drone-based deliveries also reaches them. Last but not least, patients think that information spreads by word of mouth, especially in rural areas. However, general practitioners want their patients to be informed either by pharmacies or via the mass media, but above all they want to make recommendations themselves. Therefore, general practitioners want to be informed about the drone-based medication delivery first through general practitioner-specific publications (e.g., medical journal, medical congresses, or through the ADApp project itself). Pharmacists in turn prefer an active marketing by themselves and drone manufactures.

However, participants also expressed necessary resources that come along with the drone-based medication delivery. Pharmacists and patients talked about environmental and economic factors. Pharmacists *"[...] need a higher benefit for higher costs."* They debated about when the delivery makes economic sense, but they also agreed that a drone-based delivery is useful if medicines *"[...] have a certain significance."* But not all pharmacists agreed with the view on economic factors. Although for all the statements it was important that *"[...] consulting comes first."*, some declared that *"[...] patients come first, not our economic interests."*. However,

they also talked about another economic difference: that consulting is also a competition advantage because *“[...] that´s the difference between what [mail-order pharmacies] don´t understand. With them, it´s all about the price. But we are health professionals. We are not a pill distributer. That´s the crucial difference.”* The patients wondered *“[...] how much it will be used.”* But they also commented that *“the more it is used, the cheaper it becomes. [...] and the more common it is, the higher the acceptance will be.”* Patients also want to have uniform regulations otherwise it is more confusing than meaningful when all general practitioners and pharmacies do it differently. Political commitment and general practitioner commitment seems indispensable for pharmacists and patients: *"You have to convince general practitioners of that, too, because otherwise it doesn't work."* (patients). Patients also raised another important issue: the technical requirements for using such technology. They stated out the problem of network access in rural areas. But especially network access and Wi-Fi are important prerequisites for the use of the app. Next to these requirements, all FG discussed about organizational resources such as enough space for the drone to land (patients). The patients also noted that general information and education to people is necessary so that the *"[...] people see the sense behind it, what is behind such drones."* Another organizational issue is that in case of immobility cooperation with nursing services are required for supporting the handover. Thus, nurses or at least relatives must be at the patient’s house so that the patient receives the medicines (patients, nurses). Moreover, there is a requirement of cooperation between pharmacists and outpatient care service (nurses). A nurse said: *"But from a pandemic standpoint, it worked. It had to work. We all pulled together, pharmacy and us. And then it works."* However, general practitioners like to have more field reports respectively pilot projects that test for functionality in reality, which in turn would show necessities and would increase their general trust. Pharmacists seem to prefer a user-centered approach that works from bottom to up: *"The reason I like this initiative is because it comes from the pharmacy community, from research, and not directly from the top. Anything that comes from the top is then not quite realistic."*

Concerns

Participants consistently expressed feelings and concerns when talking about the drone based medication delivery. Seven issues have been discussed which were not only triggered by the closing questions but occurred constantly in the course of the FG. A big issue highly discussed by all sample groups was the human aspect. Within the patient FG, the issue of their COVID-19 disease was most visible when talking about concerns of drone-based medication delivery. Some of the patients felt stigmatized during their disease through that may stand in connection with a disease. This was also an issue when they talked about the drone-based delivery. They wondered *"what if I am really sick and everyone around me is healthy? And now someone sees that I'm being supplied with a drone there, you're also stigmatized. This is how I have experienced it. The police was standing in front of our door, the health department was standing in front of our door, and the others go to work and think, "Why is the police car here?" You know, I was crying. Maybe it's easier in big cities, because you're running around anonymously. But how is it really when you are now supplied by such a drone and the whole world knows: You caught Corona."* In this vein, they also are afraid of data misuse which has an impact on their feeling of security, especially when having an infection.

A fertile debate was carried out about the human component that some of the patients and general practitioners mentioned as a deficiency. *"I felt that firsthand with my mother, who lives in the country. We were all flat on our backs and my mother couldn't even get up. I didn't know what to do. I phoned around with the health department lying in bed with a fever and no one took care of my mother. But then, fortunately, a general practitioner agreed and went to see her on Sunday. And now she always says: "The general practitioner saved my life". It wasn't about the medication at all. Do you know what it was about? It was about feeling that someone was helping her. This human component...I also have a cell phone and I do a lot on the Internet*

and also I am ordering things, but I'm missing a bit of humanity." (patients). The general practitioners discussed the missing human component as well. They meant that *"[...] it's important that the medicines get to the patient. I know that patients are very grateful when there is a person handing over the meds, that is, a human being at the door and not some drone landing in front of the building."* (general practitioners).

All participants agreed that the lack of digital competence, especially for older people, could be one of the biggest problems. *"I think it's difficult. Many patients don't even have an iPhone or a smartphone. So, they can't handle the technology. And then it wouldn't be feasible at all. Especially in rural areas, where the logistical connection is difficult, the older people can't handle the technology. And then the drone is not quite operational."* (general practitioners). General practitioners also noted that when drugs are very fragile, they *"[...] wouldn't rely on it being picked up reliably [by patients].",* which was also matter of concern for the nurses: *"And then there's just the question of whether the patient can assess that? For example, how much space does the drone need to land?"* Another important point nurses mentioned was that *"the older people are no longer fine-motored. How do they get to these medicines well?"* Moreover, they were concerned *"[...] that this ping-pong communication that takes place at the counter, that would actually only work one-sided. That could lead to problems."* Regarding communication problems they also talked about *"[...] barriers, if the user is not German-speaking now."* Pharmacists believe that *"the customer won't take one more app to do it, too"*. Nurses confirm this assumption by saying that medical apps sometimes are too complicated in managing such a huge amount of prescriptions. Patients also confirmed this by discussing about the focus on cell phones. One patient said: *"That pisses me off, everything is only available via the cell phone. If the cell phone is gone, what then? If you've misplaced it or forgotten it or it's broken, then you don't have anything anymore."* However, pharmacists also said: *"I don't think it matters how [the patients] get [the medicine]."* In contrast, patients thought about the impact on work conditions and were concerned about the loss of jobs through drones.

All participants also stated that *"[...] most people don't have enough experience with drones."* (patients). They talked about their own inexperience as well as the general lack of sufficient knowledge about drones including the capacity of the load, quantity of medications, charging, flight range, and landing procedure. This was particularly evident in the following commentary: "*Our pharmacies drive really fast and are very accessible and by phone we have good communication."* (general practitioners). Additionally, nurses wondered about *"what medicine is urgent, but not so urgent that the emergency general practitioner has that with him? And if it's really urgent, then maybe he will take the patient with him to the hospital."*

An issue that also all participants discussed was the economical aspect. *"Because of the economics, I can imagine that the use cases where that's really limited to those emergencies are pretty small and that probably then tends to lead to pharmacies maybe not taking the system because it's just too expensive."* (patients). *"It's also a question of what it costs afterwards."* (general practitioners). Nurses also noted that also other *"[...] companies have been involved with the delivery of parcels, for example. And they've stopped it now after two or three years because it was too complicated."* (nurses).

In addition to human or economic related aspects, all sample groups also talked about organizational and infrastructural aspects. They expressed skeptics and ambiguity about patients' involvement in processes, interactions in cities, and the level of patient participation in communication. They wondered where they include the patient: *"How do I include him in a way that doesn't make the customer feel over-advised or that he then jumps off and says: I don't want to, it's not important to me."* (pharmacists). Pharmacists have concerns about the space in and the location of their pharmacies: *"What do I do when I have an airport nearby?"* Patients talked much about their concerns of how to get their medicine if they live in the city and/or are immobile. They are also concerned about the possibility of the medicines being stolen in the city. Nurses cannot imagine how medicines can be delivered in an apartment house in the city and reached by patients, especially in case of immobility, too. *"Or if you just live in an area*

where it's difficult to impossible to get that done with the drone." (general practitioners). Nurses discussed most about the implementation of drone in the ambulatory nursing service: *"The handover, I consider this to be a problem, with us at least, in the ambulatory area."* However, the patient focus laid on the problem of poor or no access to network and Wi-Fi, mainly in rural areas. *"We have Wi-Fi to some extent, but you can't really make phone calls either. I don't know how it is in rural areas. There are still so many dead spots where nothing works."* (patients)

All sample groups talked also about legal aspects: *"If it fails, it will fail on that."* (pharmacists). When talking about this issue, participants wondered about the loss or crashes of the drones (pharmacists, patients), the rights of flying over other people's properties (patients) as well as insurances (pharmacists): *"[...] and if then my anesthetics lies in my neighbors garden and the small child takes the medicines. Who is then the one with the insurance?"* Generally, pharmacists, nurses, and general practitioners were afraid of delivering anesthetics because *"[...] that is already dangerous. Carrying with me 3000, 4000, 5000 euros I would not want to drive out with the drone at the current state"*. (pharmacists). General practitioners additionally noted *"If someone else gets the drone, then it's difficult."* Nurses wondered *"[...] how is the shipment protected from unauthorized unloading?"* Moreover, nurses questioned *"[...] what happens, for example, if the drone injures someone. Or I'm elderly and can't get out, but outside is my grandson. All of a sudden, the drone arrives. The grandchild doesn't get it that he has to get out of the way now, and the drone hurts the child. What happens then? So that's always the big question now with autonomous driving, when the car does run over someone."* Besides these concerns, pharmacists wondered if they need a shipping / trading permission when delivering medicines by drone. In a broader context, general practitioners are concerned that the clarification of airspace will become too complex.

Further, the participants considered drone-related factors. Fitting the law framework, pharmacists, nurses, and patients are concerned that the drone could injure patients, children, and dogs. In addition to questions about the rights of flying over other people's property, general

practitioners and patients said this increases the risk that drones could be destroyed. *"There are drone haters who, after all, don't know that a person is waiting for their medicine right now."* (patients). Pharmacists, nurses, and patients have concerns about the reliability of delivery due to darkness or weather dependence of drones. Moreover, patients find it *"[...] a great solution that you get something at all, but I don't know if that can be slowed down once it's up and running. That it will take on a life of its own, that other providers or companies will take it up and I don't know if you can stop it so that there really isn't drone traffic in the air all of a sudden."* This matches the patients' worry about the noise of drones and the impairment caused by drones for people and animals. Pharmacists also have completely different questions about the availability of the drones and its ownership which has implications for their daily work. Another issue pharmacists talked about concerns accessibility to the special service. They noticed that the drone-based medication delivery *"[...] would be a nice story, but the difficulty is really getting through."*

Despite many proposed solutions, a major issue the pharmacists struggled with was the documentation of the identification of the patients when receiving the delivery. *"I've also thought about how I actually want to document that the person, the patient, has accepted it. That's the most difficult thing, I think, isn't it?"*

Table 3. Users´ issues assigned to TOE factors per category.

	**organizational factors**	**environmental factors**	**technical factors**
**knowledge-base**	• lack of users´ expertise about drones and medication apps		
**problems**	• delayed / limited medication supply	• limited support<br>• pandemic regulations<br>• economic efficiency	• digital isolated solution
**usefulness**	• limited mobility	• fewer cars and papers	

	• rapid medication availability<br>• emergency and disaster<br>• regular medications<br>• reliefs<br>• retention reductions<br>• logistic reduction<br>• delivery at night<br>• flexibility	• contactless delivery	
**usability: general**	• speech recognition<br>• regular medications<br>• data sovereignty and security<br>• control over delivery process (plannability)		• additional device<br>• interface integration (only automated solutions without analog steps)<br>• scalable drones for different use cases
**usability: communication**	• consultation options<br>• reconciliation options<br>• medication plan and intake information<br>• note functions for delivery	• drone labeling	
**usability: process**	• shipment tracking (including delivery location and navigation)<br>• bilateralism<br>• order summary<br>• infrastructure (adaptable to daily routines)		• photo function for physical prescriptions<br>• availability and charging status of drones
**usability: handover**	• identification<br>• payment via app		• winch or landing
**accessibility**	• information through pharmacists and general practitioners, broad media, health insurance companies<br>• space for landing<br>• need for general information and education/training<br>• corporations<br>• user-centered approaches and pilot testing	• economic sense<br>• competition advantage through consulting via app<br>• uniform regulations<br>• political and general practitioner commitment	• network access

**concerns**	• stigmatization through visible delivery<br>• lack of humanity<br>• lack of users´ digital competences and fine-motoric skills<br>• communication barriers<br>• app over floating<br>• lack of users´ experience and knowledge about drones<br>• space for drones<br>• handover in case of immobility and apartment house<br>• lack of accessibility<br>• identification during handover	• lack of economy<br>• lack of regulations<br>• loss, crashes, and injuries with drones<br>• drone destruction<br>• regulation uncertainty (e.g., flying over people´s properties, insurances. anesthetics, theft)<br>• misuse<br>• ownership	• poor network access, especially in rural areas<br>• lack of resistance to climate conditions<br>• drones´ noise

Discussion

The application of drones in the supply process of medication are generally supported by the participants of this focus group discussion. They described a differenced picture of their expectation regarding human drone interaction and the implementation in the app based supply chain.

Despite the enormous focus and benefit of drones in healthcare, and especially in the delivery of medical supplies [7], it is surprising that drones are not yet part of the health care system. Besides legal conditions, the most important factor affecting the use of drones is user acceptance [7]. To date, reviews and studies focus on social acceptance of civil drones or the benefit and challenges of drones in society [5,7,9,10,13]. Using “user-centered-designs” is one important design to develop user acceptance positively and to further increase acceptance of drones in health care settings. Thus, the present study is embedded in a mixed-methods study

design and is intended to provide needs and requirements of users in a specific scenario of drone-based medication delivery amid the ongoing COVID-19-pandemic situation.

Generally, the present study shows problems of perceived limited support and delays in medication in the context of the pandemic situation. In this vein, drones are considered useful in cases of limited mobility, time-critical medicines (rapid availability), or of emergency (e.g., pandemic situations) and disaster situations (e.g., flooding), but also for the delivery of regular medications (e.g., in the care of chronic diseases). However, the study demonstrates that very few of the respondents had any knowledge and competence about drones (16.7%) or medication apps (32.2%). According to results of Eißfeld et al. [13], a general technical interest and knowledge about drones play an important role for acceptance. In other words, the better people are informed about benefits and challenges of drones, the more they accept them. It can be concluded that concerns and usability aspects, especially the handover, accounted for the majority of discussions over all focus groups of all five categories (problems, usefulness, usability, accessibility, and concerns). With regard to the low level of knowledge about drones and medication apps, it is not surprising that concerns make up for the most discussion [13]. However, these results imply the need for general information and education to increase technical knowledge and competence. According to the TAM, the attitude toward using a technology is dependent on perceived ease of use and perceived usefulness [14,15]. Thus, not only general clarifications of drones are necessary, rather providing drone technologies and systems for a specific scenario in health care is able to shape experience in a positive way. The ongoing ADApp study will provide insights whether the iterative involvement of users during technology development will increase user acceptance. At this point, responses of users can be implemented in the present development of an app-assisted drone-based medication delivery.

Issues influencing user acceptance

Organizational, environmental, and technical factors are considered to affect the use and performance of drones whereby usability and design of drones affect them most [7,9]. According to these TOE factors, the present study identified needs and requirements of users, which can each be assigned to these TOE factors (see **Table 3**). The frequency of discussed issues show a certain heightening of users´ needs. Just in one review it was shown that it became apparent that usability aspects contribute to the most proportion of discussion which is also present in our results [7].

App-related issues

The communication aspect seems to be more important when people are in need. In a previous study using an AED delivery scenario, it was found that interaction with the dispatcher gave the participants a sense of security that results in a positive effect on compressions [36] (see **Table 4** for a comparison between results of a previous scoping review [12] and the present study). Thus, one important issue influencing the acceptance of drones in the delivery of medicines is the need of consultation features via app. It seems that when humanity decreases, technologies in turn need arrangements to meet the need of more relatedness [37]. These results show the benefit of user-centered studies since the consulting aspect has been often overlooked so far [12]. Another important usability aspect regarding communication is that all information and the entire process have to be continuously digital without analog steps in between. Especially the pharmacists emphasized that they would not tolerate a stand-alone solution outside pharmacy technology otherwise it makes more work and it would be not attractive for them. This implies that new technologies involving different parties have to be integrated in and connected with existing health care services in an automated manner (IoMT).

Another important organizational factor that has to be included in a pharmacy-drone-app is a shipment tracking for a transparent delivery process. These issues were also identified

in a previous scoping review [12] (see **Table 4**). It was found that users expressed uncertainty and anxiety about the lack of knowledge of the direction the drone comes from, the drone's look and sound, as well as the landing and handover process in an AED delivery scenario [36,38,39]. This was the most identified response of users (measured in terms of the number of studies) [12]. Thus, users need the information about the delivery process for reducing uncertainty and concerns, which is especially useful in situations where people are in need. From a basic psychological perspective taking needs into account, these results can be assigned to an agglomeration of autonomy, relatedness, and competence, i.e., the need of having meaningful choices in an effective manner while feeling connected to others [37]. Sufficient information about the delivery process could make the user feeling safe, connected, and able in handling the process. It provides a feeling of agency, overall. Another important issue discussed was the point of bilateralism, i.e., either the general practitioners make out an e-prescription or the patient requests an e-prescription. Thus, an app like the pharmacy-drone-app should include features of a bidirectional process that simplifies the process. This might useful in case of chronic diseases to facilitate accessibility to patients and to reduce costs. In this sense, there is a need for finding a balance between the accesses to new technology for the most vulnerable and maintaining of economic interests.

Regarding handover, the most discussed organizational factor was the identification during the receipt of the medicine. Especially pharmacists discussed the most about the handover process. They expressed concerns about the manner of documentation that the medicine has actually been delivered / received, which is most important when transporting anesthetics.

However, participants expressed a high discrepancy between the usefulness of drones, especially in rural areas, and the lack of network connectivity in rural areas, digital literacy, and fine-motoric skills, especially when getting older. Patients and nurses suggested speech recognition features within the app to order the medicine in a voice-controlled manner and to get an announcement according to delivery status, which makes it easier for people with less digital

literacy or impairments. Above all, patients were upset about the fact that every technology can only be operated with smartphone, producing hence an app flood and that in case of loss all data are gone. However, beside a cloud-based solution, patients suggested an additional device (homedot) like an Alexa dot or Homepod but only for medical matters. Another discrepancy is that drones are useful in cases of limited mobility but not useful in case of immobility when a patient cannot leave the house or live in a region where it is difficult to impossible to get the medication by drone (e.g., in cities). Regarding this concerns, participants expressed different ideas. One important issue is the coordination between the ambulatory nursing service and the drone-based delivery so that either the medication would be delivered to the office of nursing service or during the visit at patient´s house.

Drone-related issues

A big issue highly discussed by patients was their experiences of stigmatization during COVID-19 disease. They expressed concerns about the visible and noisy delivery of medica-tions with the drone. Controversially, they talked about risks that drones could be destroyed by other people who do not appreciate drones flying over their properties and possibly being una-ware about the importance of the drone. However, patients prefer to label drones as medical drones since the arrival of medicines in emergencies seems more important than the stigmati-zation. Moreover, the technical development of drones should be directed toward reducing noise. The stigmatization and noise pollution were expressed as concern. A recent study of Eißfeld et al. [13] revealed that concerns about noise have the strongest impact on acceptance. However, results of the present study showed that participants believe that acceptance of drones would increase when the service of drone-based delivery will become more common, when more user-centered approaches and field reports respectively pilot projects are performed that test for functionality under real life conditions. The participants mentioned that such projects show necessities and increase their general trust because they have also concerns about the

abuse of drones to criminal purposes. Another study has also shown that participants mentioned such concerns (91%) [13]. Thus, more user-centered research on drones with concrete scenarios are necessary to improve acceptance and trust.

Studies have shown that the physical contact with the drone is one of the biggest user concerns [36,38,39]. One solution could be using a winch that reduces direct physical contact with the drone. Results of the present study also suggest that participants prefer the handover of medications via winch, although landing was a discussed alternative, which is implementable in rural areas or at institutions with a central landing area (see **Table 4**).

However, some other factors influencing the performance of drones in health care are concerns about the lack of the drone´s resistance to climate conditions, especially at night. In the future, drones should be developed to be more resistant to harsh conditions. Lights at the drone could be a solution for at night deliveries [12]. However, patients need the commitment that the medication is getting to them as fast as possible. Above all, legal restrictions and questions of insurances make it difficult to test drones in real context. That is the reason why pilot projects are conducted mostly in Africa. There is clearly a need of studies in Europe to investigate the role and acceptance of drones in European health care because only in this way laws may be changed and adapted and the adaption from low and middle income countries in Africa is very limited, but the economic perspective is truly better in the health care systems of the high income countries To reach this the first important step is, that users be aware of solutions to their problems and should accept new technologies before superordinate structures can be changed. Moreover, health workers must learn about how to integrate drones into health supply chains by involving them into the development of such technologies. However, this could be a fact that is of no difference regarding Africa and Germany since a case study found that users expressed that donors and operators "do what they want, and not what we want" [11].

Table 4. Comparison of results of previous scoping review [12] and present study.

Scoping Review [12]		Present Study		Scoping Review [12]		Present Study
**Perceived Support**		**Usability**		**Concerns and Wishes**		**Concerns**
app-related						
interaction with dispatcher	≙	consultation				
live-video streaming	≙	identification handover				
drone-related						
				injury by drone	=	injury by drone
				lack of resistance to climate conditions	=	lack of resistance to climate conditions
		shipment tracking, delivery status	≙	uncertainty about drone´s arrival		
		drone labeling as medical drone	≙	reflective tapes or lights for marking location of arriving	≙	drone destruction

Limitations

When interpreting the results, several methodological limitations have to be taken into account. The sample corresponds to the typical comparative figures with regard to age and gender distribution, but cannot be classified as representative due to the small number of participants. However, due to the first saturation of the data material, a certain generalizability of the results can be assumed [33]. The participating actors from the health sector had a fundamental interest in new topics and in the issue itself. However, the risk of a possibly “positive selection” is not detected, as the topic area of drones and their use for medical deliveries was mostly unknown to the health care actors. Thus, positions that consistently reject drones in the context of medical supply delivery were just as little represented as those that refrained from participating in the

focus group for other reasons. Reasons for this could be, among others, a general negative attitude toward additional efforts due to time resources, heavy workloads, or other thematic priorities. Nevertheless, it can be assumed that the results and interpretative derivations of the present study could, under certain conditions, also be valid for other parts of Germany as well as for other areas in which the use of drones in health care is in focus (e.g., in palliative care). Regarding the linguistic aspect of the contribution, it should be mentioned that all focus groups were conducted and documented in German. Even though the quotations used in this paper were translated into English with the help of a native speaker, a literal reproduction is not always given since accessibility and readability are of importance, too. Any slight shift in meaning was checked using back-and-forth-translations.

Conclusion

As this study illustrates, COVID-19-patients, pharmacists, nurses, and general practitioners think that drones offer an alternative option for transporting medicines in order to help people in need to improve access to medical supply, especially in rural areas and for people with limited mobility. However, they described the lack of economic efficiency of digital solutions and the increase in isolated solutions. At this point, connecting an app with a drone system seems useful for participants to know the location of handover and to adjust the medicine receipt. Thus, participants emphasized that the pharmacy-drone-app must be as lean and simple as possible (e.g., with speech recognition and medical voice-controlled homedots), must facilitate processes and routines (e.g., software integration, plannability, shipment tracking), has to be completely digital, must enable control (e.g., data sovereignty, handover documentation), and should include consultation and reconciliation features. There is clearly a need of user-centered studies investigating the role and acceptance of drones in health care to learn about how drones can be integrates into the health supply chain system. User-centered designs have

the added value that users find the solutions to their problems on their own in exchange with each other and thus have a higher impact in technical implementation.

Acknowledgments

We would like to thank the ADApp-Team for exchange and discussion of the focus group guide and for evaluating the results of focus groups for further technical development. A special thank goes out to Prof. Dr. Axel Schlitt from the Paracelsus Harz-Clinics Bad Suderode, the Medical Journal (Deutsches Ärzteblatt), the Pharmacy-ADHOC Journal (Apotheke-Adhoc), and the DBfK - The German Nurses Association (Deutscher Berufsverband für Pflegeberufe DBfK e.V.) for their help with participant recruitment. We would also like to thank Anne-Marie Lachmund who was responsible for proof-reading and stylistic adjustments.

References

1. Elo S, Kyngäs H. The qualitative content analysis process. J Adv Nurs. 2008; 62:107–15. doi: 10.1111/j.1365-2648.2007.04569.x PMID: 18352969.

2. Berger K, Riedel-Heller S, Pabst A, Rietschel M, Richter D. Einsamkeit während der ersten Welle der SARS-CoV-2-Pandemie – Ergebnisse der NAKO-Gesundheitsstudie. Bundesgesundheitsbl. 2021; 64:1157–64. Epub 2021/07/29. doi: 10.1007/s00103-021-03393-y PMID: 34327541.

3. Richter D, Riedel-Heller S, Zürcher SJ. Mental health problems in the general population during and after the first lockdown phase due to the SARS-Cov-2 pandemic: rapid review of multi-wave studies. Epidemiology and Psychiatric Sciences. 2021; 30:e27. Epub 2021/03/09. doi: 10.1017/S2045796021000160 PMID: 33685551.

4. Chamola V, Hassija V, Gupta V, Guizani M. A Comprehensive Review of the COVID-19 Pandemic and the Role of IoT, Drones, AI, Blockchain, and 5G in Managing its Impact. A Comprehensive Review of the COVID-19 Pandemic and the Role of IoT, Drones, AI, Blockchain, and 5G in Managing its Impact. IEEE Access. 2020; 8. doi: 10.1109/access.2020.2992341.

5. Rosser JC, Vignesh V, Terwilliger BA, Parker BC. Surgical and Medical Applications of Drones: A Comprehensive Review. JSLS. 2018; 22. doi: 10.4293/JSLS.2018.00018 PMID: 30356360.

6. Scalea JR, Pucciarella T, Talaie T, Restaino S, Drachenberg CB, Alexander C, et al. Successful Implementation of Unmanned Aircraft Use for Delivery of a Human Organ for Transplantation. Ann Surg. 2019. Epub 2019/10/28. doi: 10.1097/SLA.0000000000003630 PMID: 31663974.

7. Hiebert B, Nouvet E, Jeyabalan V, Donelle L. The Application of Drones in Healthcare and Health-Related Services in North America: A Scoping Review. Drones. 2020; 4:30. doi: 10.3390/drones4030030.

8. Poljak M, Šterbenc A. Use of drones in clinical microbiology and infectious diseases: current status, challenges and barriers. Clin Microbiol Infect. 2020; 26:425–30. Epub 2019/09/28. doi: 10.1016/j.cmi.2019.09.014 PMID: 31574337.

9. Rejeb A, Rejeb K, Simske S, Treiblmaier H. Humanitarian Drones: A Review and Research Agenda. Internet of Things. 2021; 16:100434. doi: 10.1016/j.iot.2021.100434.

10. Wang N, Christen M, Hunt M. Ethical Considerations Associated with “Humanitarian Drones”: A Scoping Literature Review. Sci Eng Ethics. 2021; 27:1–21. doi: 10.1007/s11948-021-00327-4.

11. Wang N. “Killing Two Birds with One Stone”? A Case Study of Development Use of Drones. 2020 IEEE International Symposium on Technology and Society (ISTAS). ; 2020. pp. 339–45.

12. Stephan F, Reinsperger N, Grünthal M, Paulicke D, Jahn P. Human drone interaction in delivery of medical supplies: A scoping review of experimental studies. PloS One. 2022; 17:e0267664. Epub 2022/04/28. doi: 10.1371/journal.pone.0267664 PMID: 35482656.

13. Eißfeldt H, Vogelpohl V, Stolz M, Papenfuß A, Biella M, Belz J, et al. The acceptance of civil drones in Germany. CEAS Aeronaut J. 2020; 11:665–76. doi: 10.1007/s13272-020-00447-w.

14. Kothgassner OD, Felnhofer A, Hauk N, Kastenhofer E, Gomm J, Ryspin-Exner I. TUI: Technology Usage Inventory: Vienna: FFG; 2012.

15. Davis FD. Perceived Usefulness, Perceived Ease of Use, and User Acceptance of Information Technology. MIS Quarterly. 1989; 13:319. doi: 10.2307/249008.

16. Ärzteblatt.de. Automatisierte Medikamenten-transporte per Drohne [updated 2021; cited 18 May 2021]. Available from: https://www.aerzteblatt.de/nachrichten/122802/Automatisierte-Medikamententransporte-per-Drohne.

17. Kelland K. Drones to deliver vaccines, blood and drugs across Ghana. Reuters 2019 [cited 18 May 2021]. Available from: https://www.reuters.com/article/us-health-vaccines-drones-idUSKCN1S0175.

18. Drones could speed up HIV tests in remote areas. Reuters 2016 [cited 18 May 2021]. Available from: https://www.reuters.com/article/us-malawi-hiv-drones-idUSKCN0XH1ZN.

19. Werber C. Rwanda Is Using Drones To Deliver Blood Donations To Remote Health Centers. The Huffington Post 2016 [cited 18 May 2021]. Available from: https://www.huffpost.com/entry/drone-delivery-service-launched-in-rwanda_n_5800f353e4b0162c043b7739.

20. Africa’s first humanitarian drone testing corridor. UNICEF 2017 [cited 18 May 2021]. Available from: https://www.unicef.org/innovation/drones/africa-first-humanitarian-drone-corridor-malawi.

21. Farao J, Malila B, Conrad N, Mutsvangwa T, Rangaka MX, Douglas TS. A user-centred design framework for mHealth. PloS One. 2020; 15:e0237910. doi: 10.1371/journal.pone.0237910 PMID: 32813711.

22. Hevner A. A Three Cycle View of Design Science Research. Scandinavian Journal of Information Systems. 2007; 19. Available from: https://aisel.aisnet.org/sjis/vol19/iss2/4.

23. Altman M. Design Thinking in Health Care. Preventing Chronic Disease. 2018; 15. doi: 10.5888/pcd15.180128.

24. Roberts JP, Fisher TR, Trowbridge MJ, Bent C. A design thinking framework for healthcare management and innovation. Healthcare. 2016; 4:11–4. doi: 10.1016/j.hjdsi.2015.12.002.

25. Robra-Bissantz S, Strahringer S. Wirtschaftsinformatik-Forschung für die Praxis. HMD. 2020; 57:162–88. doi: 10.1365/s40702-020-00603-0.

26. Oort FJ, Visser MRM, Sprangers MAG. Formal definitions of measurement bias and explanation bias clarify measurement and conceptual perspectives on response shift. J Clin Epidemiol. 2009; 62:1126–37. Epub 2009/06/21. doi: 10.1016/j.jclinepi.2009.03.013 PMID: 19540722.

27. McCurdie T, Taneva S, Casselman M, Yeung M, McDaniel C, Ho W, et al. mHealth Consumer Apps: The Case for User-Centered Design. Biomedical Instrumentation & Technology. 2012; 46:49–56. doi: 10.2345/0899-8205-46.s2.49.

28. Tong A, Sainsbury P, Craig J. Consolidated criteria for reporting qualitative research (COREQ): a 32-item checklist for interviews and focus groups. International journal for quality in health care: journal of the International Society for Quality in Health Care. 2007; 19:349–57. doi: 10.1093/intqhc/mzm042 PMID: 17872937.

29. Helfferich C. Die Qualität qualitativer Daten. Manual für die Durchführung qualitativer Interviews. 4th ed. Wiesbaden: VS Verlag für Sozialwissenschaften / Springer Fachmedien Wiesbaden GmbH Wiesbaden; 2011.

30. Kothgassner OD, Felnhofer A, Hauk N, Kastenhofer E, Gomm J, Ryspin-Exner I. TUI: Technology Usage Inventory: Vienna: FFG; 2012.

31. Bohnsack R, Przyborski A, Schäffer B, editors. Das Gruppendiskussionsverfahren in der Forschungspraxis. 2nd ed. Opladen: Budrich; 2010.

32. Schulz M, Mack B, Renn O, editors. Fokusgruppen in der empirischen Sozialwissenschaft. Von der Konzeption bis zur Auswertung. Wiesbaden: Springer VS; 2012.

33. Lamnek S. Gruppendiskussion. Theorie und Praxis. Weinheim: Beltz; 2005.

34. Kuckartz U. Qualitative Inhaltsanalyse. Methoden, Praxis, Computerunterstützung. 3rd ed. Weinheim, Basel: Beltz Juventa; 2016.

35. Gläser J, Laudel G. Experteninterviews und qualitative Inhaltsanalyse. Als Instrumente rekonstruierender Untersuchungen. 4. Aufl. Wiesbaden: VS Verl.; 2010.

36. Sanfridsson J, Sparrevik J, Hollenberg J, Nordberg P, Djärv T, Ringh M, et al. Drone delivery of an automated external defibrillator - a mixed method simulation study of bystander experience. Scand J Trauma Resusc Emerg Med. 2019; 27:40. Epub 2019/04/08. doi: 10.1186/s13049-019-0622-6 PMID: 30961651.

37. Peters D, Calvo RA, Ryan RM. Designing for Motivation, Engagement and Wellbeing in Digital Experience. Frontiers in Psychology. 2018; 9. doi: 10.3389/fpsyg.2018.00797.

38. Rosamond WD, Johnson AM, Bogle BM, Arnold E, Cunningham CJ, Picinich M, et al. Drone Delivery of an Automated External Defibrillator. N Engl J Med. 2020; 383:1186–8. doi: 10.1056/NEJMc1915956 PMID: 32937053.

39. Zègre-Hemsey JK, Grewe ME, Johnson AM, Arnold E, Cunningham CJ, Bogle BM, et al. Delivery of Automated External Defibrillators via Drones in Simulated Cardiac Arrest: Users' Experiences and the Human-Drone Interaction. Resuscitation. 2020; 157:83–8. doi: 10.1016/j.resuscitation.2020.10.006 PMID: 33080371.

Supporting information

S1. COREQ Checklist.

 Click here to access/download;Figure;Fig.1.tif

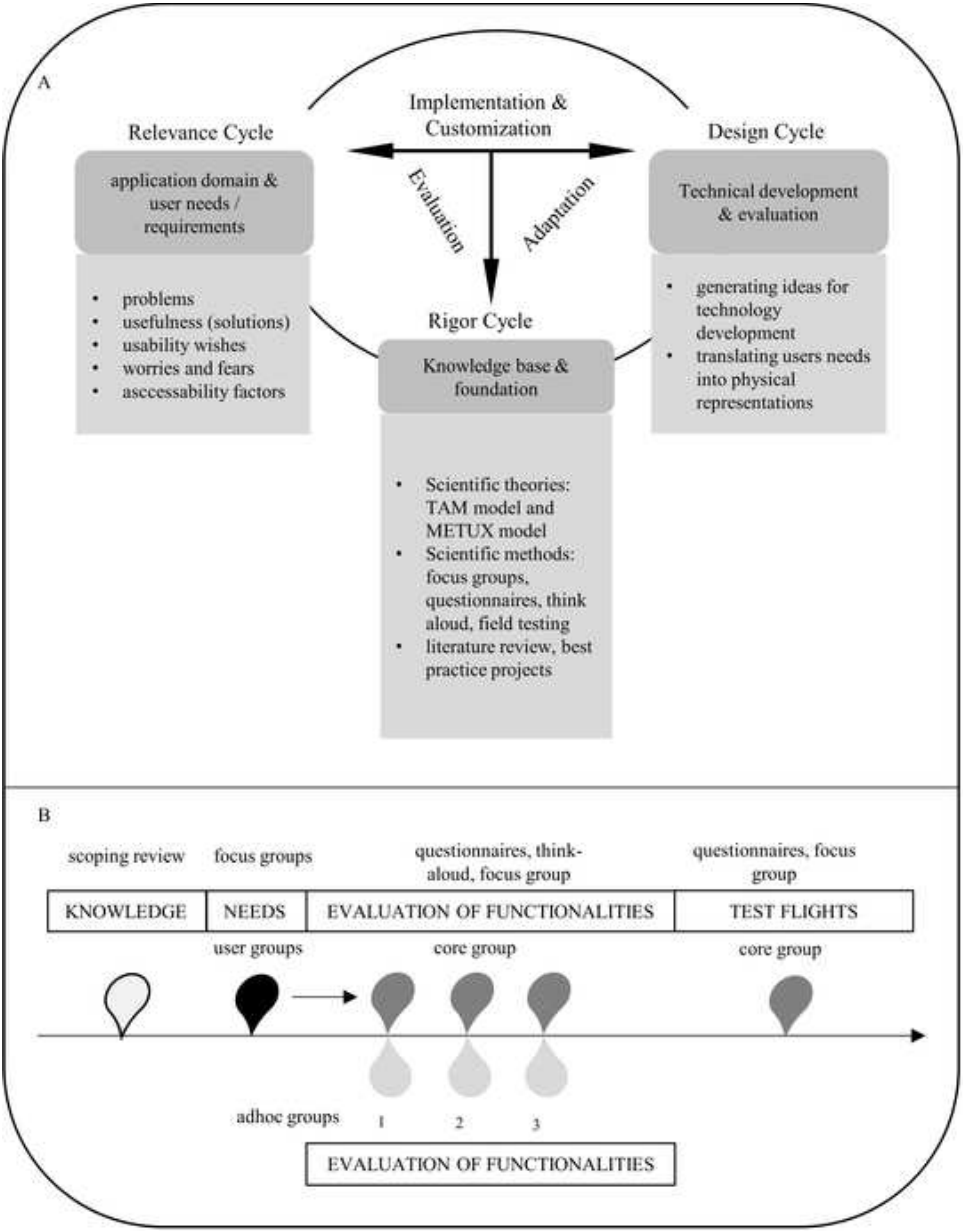

Figure 2

Click here to access/download;Figure;Fig.2.tif

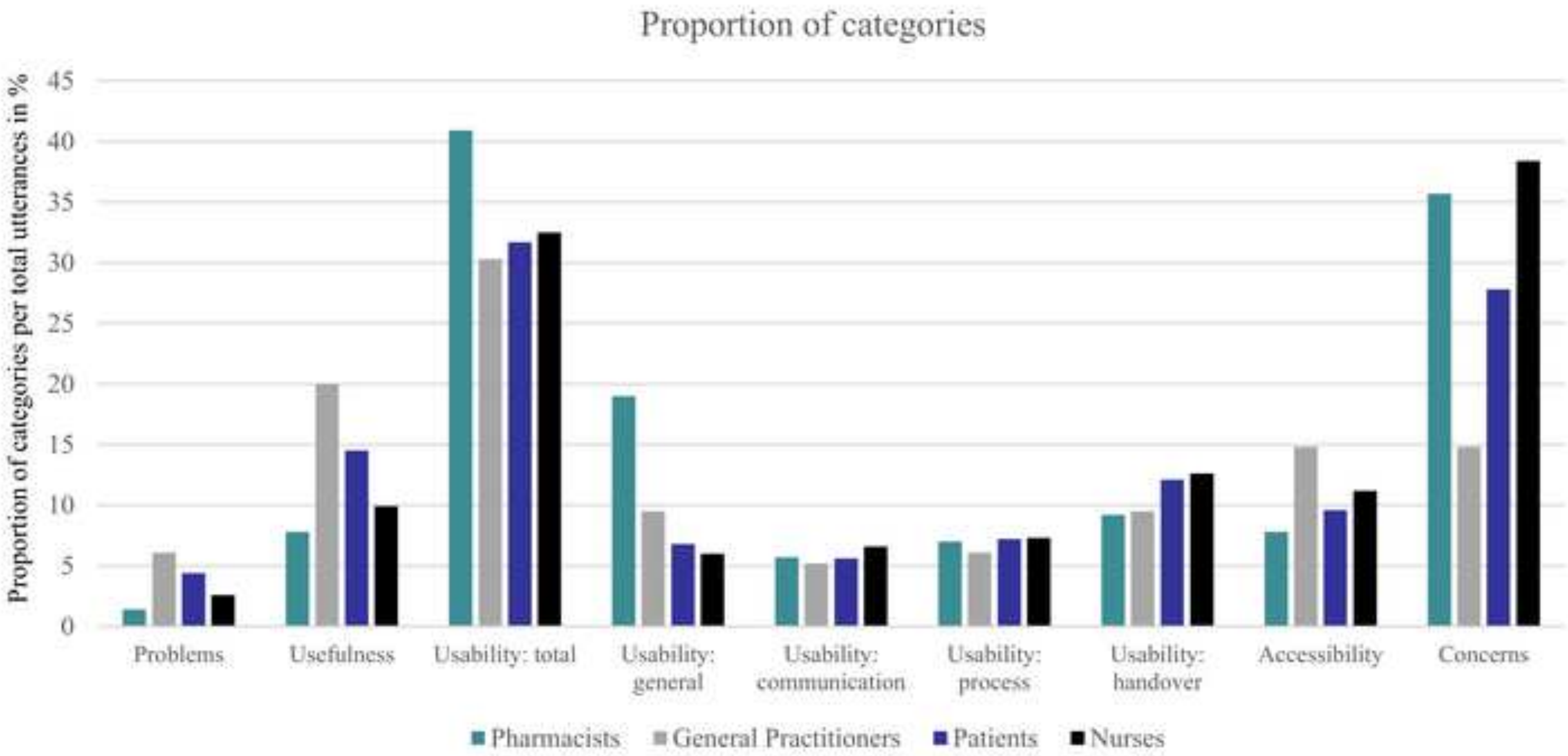

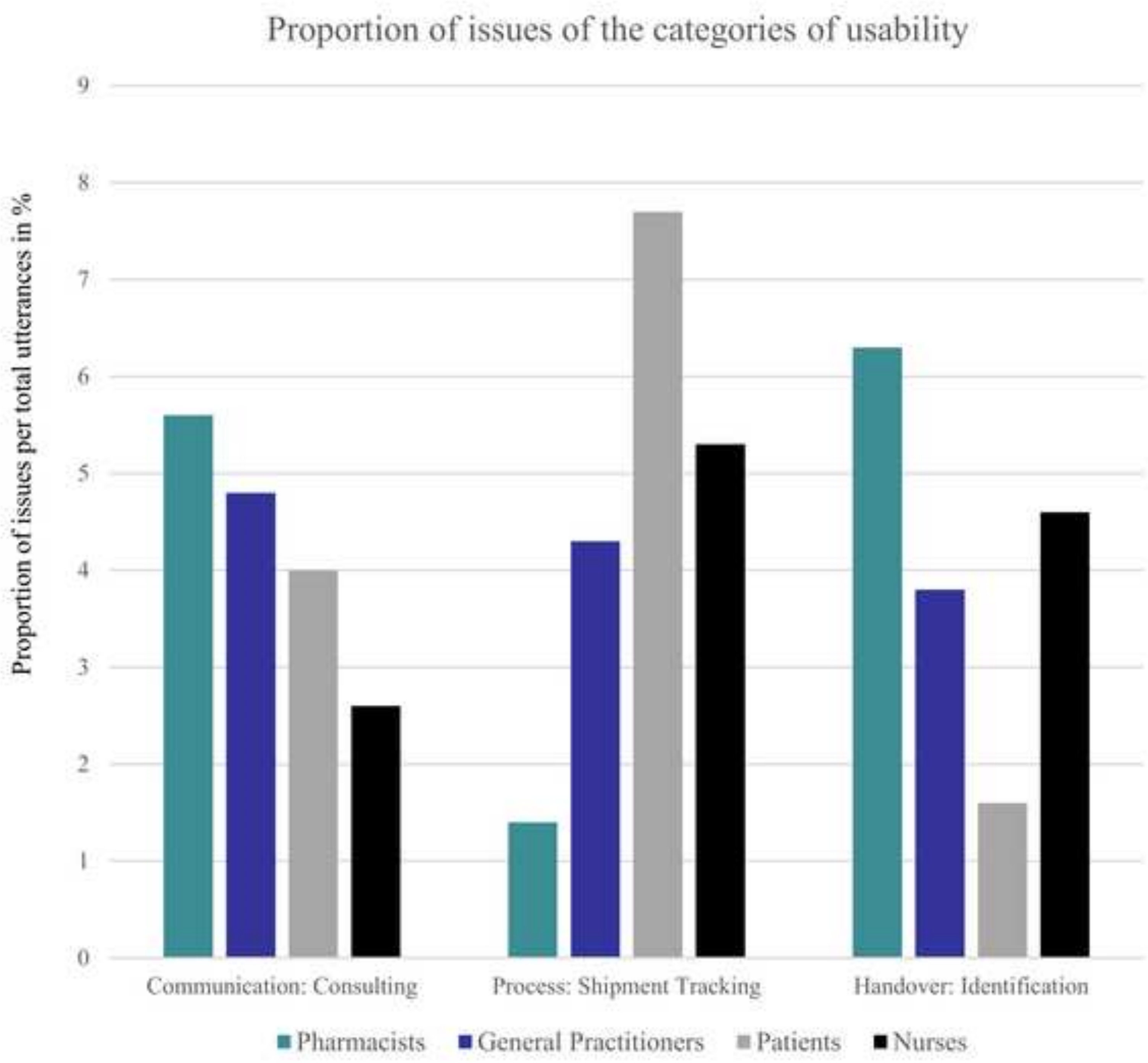
Proportion of issues of the categories of usability
Proportion of issues per total utterances in %
0
1
2
3
4
5
6
7
8
9
Communication: Consulting
Process: Shipment Tracking
Handover: Identification
Pharmacists
General Practitioners
Patients
Nurses

Click here to access/download
Supporting Information
ADApp_focus group_Appendix S1.docx
