# Supplementary material for: “Of course, drones delivering urgent medicines are necessary. But I would not use them until…” Insights from a qualitative study on users’ needs and requirements regarding the use of medical drones
Source: PLoS One. 2023 May 8;18(5):e0285393. doi: 10.1371/journal.pone.0285393 (PMC10166545; doi:10.1371/journal.pone.0285393)
Supplement: Attachment — Submitted filename: responses_to_reviewers_PONE-D-22-13044.docx [file pone.0285393.s006.docx]

**Responses to reviewers**

Manuscript Number: PONE-D-22-13044

“Of course, drones delivering urgent medicines are necessary. But I would not use them until…” Insights from a qualitative study on users’ needs and requirements regarding the use of medical drones

PLOS ONE

Dear Editors,

Thank you very much for the opportunity to submit a revised version of our manuscript. We are very thankful for the constructive comments provided by the reviewers. In the following, we address all reviewers´ comments in a point-by-point manner and hope the manuscript is now ready for publication in PLOS ONE. Changed paragraphs were marked in blue color throughout the manuscript.

Thank you very much.

Yours sincerely,

Denny Paulicke (corresponding author), on behalf of all co-authors

**Editor´s comments**

**When submitting your revision, we need you to address these additional requirements.**

**1. Please ensure that your manuscript meets PLOS ONE's style requirements, including those for file naming. The PLOS ONE style templates can be found at**

[**https://journals.plos.org/plosone/s/file?id=wjVg/PLOSOne_formatting_sample_main_body.pdf**](https://journals.plos.org/plosone/s/file?id=wjVg/PLOSOne_formatting_sample_main_body.pdf) **and**

[**https://journals.plos.org/plosone/s/file?id=ba62/PLOSOne_formatting_sample_title_authors_affiliations.pdf**](https://journals.plos.org/plosone/s/file?id=ba62/PLOSOne_formatting_sample_title_authors_affiliations.pdf)**”**

**Response:** We have checked the style templates for the title page and manuscript.

**2.** **PLOS requires an ORCID iD for the corresponding author in Editorial Manager on papers submitted after December 6th, 2016. Please ensure that you have an ORCID iD and that it is validated in Editorial Manager. To do this, go to ‘Update my Information’ (in the upper left-hand corner of the main menu), and click on the Fetch/Validate link next to the ORCID field. This will take you to the ORCID site and allow you to create a new iD or authenticate a pre-existing iD in Editorial Manager. Please see the following video for instructions on linking an ORCID iD to your Editorial Manager account:** [**https://www.youtube.com/watch?v=_xcclfuvtxQ**](https://www.youtube.com/watch?v=_xcclfuvtxQ)**.**

**Response:** Many thanks for the feedback. The corresponding author, Denny Paulicke, has the following ORCID iD: 0000-0002-9808-215X

**3. One of the noted authors is a group or consortium [Denny Paulicke]. In addition to naming the author group, please list the individual authors and affiliations within this group in the acknowledgments section of your manuscript. Please also indicate clearly a lead author for this group along with a contact email address.**

**Response:** We had to change the corresponding author from Franziska Fink (née Stephan) to Denny Paulicke. Denny Paulicke is now the corresponding author. We indicated this in the manuscript on the title page. Both authors, Paulicke and Fink, contributed equally to the present manuscript and share the lead authorship.

**4**. **Your ethics statement should only appear in the Methods section of your manuscript. If your ethics statement is written in any section besides the Methods, please delete it from any other section.**

**Response:** Thank you for your comment. We included our full ethics statement in the Methods section.

**Reviewer 1**

**1. Is the manuscript technically sound, and do the data support the conclusions?

Reviewer #1: Yes**

**2. Has the statistical analysis been performed appropriately and rigorously?**

**Reviewer #1: Yes**

**3. Have the authors made all data underlying the findings in their manuscript fully available?

Reviewer #1: Yes**

**4. Is the manuscript presented in an intelligible fashion and written in standard English?

Reviewer #1: No**

**Response:** Thank you for your feedback. We have shortened and partly simplified the manuscript to make it more intelligible. The proofreading has been repeated to adjust the language.

**5. Review Comments to the Author**

**Thank you for the opportunity to review this paper. It is a very interesting study and the findings will have significant implications in this current era. While most sections are very well written in terms of scientific arguments, several sections are too long and should be made more concise so as not to put off the readers. Especially certain sections in the Results - please try and trim some of the quotes and also limit the number of quotes used. Consider perhaps using a tabular format to capture and present some of the quotes. Please find the rest of my comments appended.**

**Response:** Thank you very much for your review and your careful considerations. We address all your comments in a point-by-point fashion. We copied your comments from the pdf file into this document for answering.

We have refined several sections, especially the results section. We put anchor examples of participants’ feedback in a tabular format and appended it as S4: “The most important examples are presented below. More detailed and in-depth examples are provided in addition file S4.” (p. 12)

**Abstract - Methods**

**1. zoom? teams? From the methods section below, it would seem that for patients this was conducted face to face?**

**Response:** Thank you for your suggestions. We now specified how we conducted the focus group:

*“Online-based focus groups (via WebEx) were conducted with participants from different*

*disciplines: nursing, pharmacy, physicians. Focus groups with COVID-19 patients were*

*conducted face-to-face.”* (p. 2)

**2. “Actors” - Maybe use a different word - participants?**

**Response:** We replaced the word “actors” with “participants”.

**3. “n = 36 participants” - this should be noted in results**

**Response:** Thank you. We moved the statement to the result section of the abstract:

*“All interview partners (patients, pharmacists, physicians and nurses; n=36 participants).”*

(p. 2)

**4. “The focus groups were fully transcribed and analyzed for content” - how exactly analysed and what software used if any**

**Response:** We described the analysis in a more detailed way. Due to word restrictions in the abstract we cannot specify it further.

*“The focus groups were audio recorded, transcribed by a transcription-company, and coded with the help of the program “f4analyse 2” for content (Elo and Kyngäs 2008).”* (p. 2)

**Abstract - Results**

**5. Provide a bit more details about the participants and the themes. Currently results are too brief**

**Response:** Thank you for pointing this out. The section has been adapted and expanded in the abstract.

**Abstract - Discussion**

**6. “knowledge and application deficits” this was not presented in the results above**

**Response:** Thank you very much for your comment. We now included the corresponding result in the result section of the abstract:

*“Moreover, only 16.7% of participants have experiences with drones.”* (p. 2)

**Introduction**

**7. Very well written but can be made more concise. It might also benefit from some sectioning, especially when talking about then ADApp-project.**

**Response:** Thank you very much for the helpful comment. We made the introduction more concise and split it into a section with “present study”. Please refer to the whole section for a complete view.

**8. “In this vein, user acceptance issues lack of user’s skills and expertise, letting negative perception and technology immaturity hinder the use of drones (Rejeb et al. 2021).“ This sentence does not read well.**

**Response:** We reformulated the sentence and hope that it now reads better.

*“In this vein, the lack of user’s skills, negative perception about the technology as well as*

*technology immaturity hinder the use of drones (Rejeb et al. 2021).”* (p. 5).

**9. “The aim is to identify the requirements of the users and to show which conditions must prevail for implementation under pandemic conditions.” This overlaps with sentence in 126 - this paper explores - consider combining.**

**Response:** Thank you for your comment. We deleted the sentence due to redundancy.

**Methods**

**10. I wonder if this whole section can be added as an appendice, and only key points pulled out to explain the method used here/reasons.**

**Response:** Thank you for your valuable consideration. We added the section of the “ADApp Design” in the appendix (named S1). Moreover, we decided to only mention the ADApp project shortly in the Introduction and put all further information about the project in the appendix. Please check out the Appendix S1 for a complete view.

**Methods – Participants**

**11. “Between June 2021 and October 2021, we collected data from 36 participants in total. Participants were 44.4% female and 55.6% male, ranging in age from 23 to 75 years (mean 50.1 ± 13.8).” AND “The FG were conducted separately and uniquely for each role characteristics resulting in four FG: general practitioners (*n* = 8), pharmacists (*n* = 8*)*, nurses (*n* = 6), and COVID-19-patients (*n* = 14).” This should be in Results.**

**Response:** Thank you, we moved the information about participants´ demographics in the result section: “Participant’s Knowledge Base” and deleted the sentence with details about the number of participants per group because the information can be found in Table 2:

***“****Between June 2021 and October 2021, we collected data from 36 participants in total.*

*Participants were 44.4% female and 55.6% male, ranging in age from 23 to 75 years (mean 50.1 ± 13.8). According to the knowledge and competence in the handling with drones, only five participants out of 30 (16.7%) stated that they already have experiences with drones.”* (p. 11)

**Methods – Materials**

**12. Can this be made more concise, with just an overview of questions? And append the Interview guide?**

**Response:** Thank you for your consideration. We made the section more concise and appended the interview guide (see S3). The section “Materials” now reads as following:

*“The FG instrument (in German language) was developed from FS through a multi-stage*

*systematic process. DP, MG, PJ, and members of the ADApp Team critically reflected the FG instrument in the context of a quality-assuring feedback (Helfferich 2011). An expert review is one potential pretesting method to determine whether the items of the FG instrument were problematic or clear in context of its goal. There was no specific instruction given to provide feedback on items, except for a short statement about an overall FG instrument goal (Rothgeb et al. 2007). After each feedback, the FG instrument was adapted. The FG instrument consisted of nine questions. These questions were based on the Technology Usage Inventory (TUI) (Kothgassner et al. 2012; Davis 1989). Three technology-specific factors (usability, usefulness, and accessibility) and four psychological factors (curiosity, interest, skepticism, and fearfulness) of the TUI were used for question development. The FG instrument includes questions about actual problems under pandemic conditions, the knowledge and competence in the handling with medication apps and drones, the general usefulness of a drone-based delivery, the usability features in communication, delivery process (and handover), the accessibility of the application and drone delivery, as well as concerns of using such technology. The interview guide and an overview of questions assigned to category and TUI factors is provided in the additional file S3. Before starting the interview, two moderators (M1 and M2) introduced the participants to the topic and persons and provided a fictive case study.”* (p. 7-8)

**13. “The FG instrument was developed through a multi-stage systematic process and was critically reflected in the context of a quality-assuring feedback and several iteration loops (peer group: project partners) (Helfferich 2011).“ Please explain this. Who was involved in validation? Was it pilot tested? What language was it in?**

**Response:** The FG instrument was developed from the first author (FS). Multiple staff members within the ADApp project as well as the second first author (DP), the co-author (MG), and the supervisor (PJ) reviewed the FG instrument (Rothgeb et al. 2007). This expert review is one potential pretest method. Rothgeb et al. (2007) were able to show that expert review is productive in identifying question problems. Every expert determined whether the items of the FG instrument were problematic or clear. There was no specific instruction given to provide feedback on items, except for a short statement about an overall FG instrument goal. We reformulated the sentences. It reads now as following:

*“The FG instrument (in German language) was developed from FS through a multi-stage*

*systematic process. DP, MG, PJ, and members of the ADApp Team critically reflected the FG instrument in the context of a quality-assuring feedback (expert review) (Helfferich 2011; Rothgeb et al. 2007). An expert review is one potential pretesting method to determine whether the items of the FG instrument were problematic or clear in context of its goal. There was no specific instruction given to provide feedback on items, except for a short statement about an overall FG instrument goal (Rothgeb et al. 2007). After each feedback, the FG instrument was adapted.“* (p. 7-8)

**Methods – Data Collection**

**14. Can be made more concise if some details are already provided in the COREQ e.g. experience and qualifications of moderators etc.**

**Response:** Thank you very much for your comment. To ensure an appropriate application and to improve the quality of reporting of our qualitative research, we used the COREQ reporting guidelines. It should enable readers to better understand the design, conduct, analysis, and findings of our study. Therefore, we cannot omit details in the data collection section. All details provided in this and other sections have to appear in the manuscript and in the checklist as well to provide qualitative reporting within our manuscript. We nevertheless hope that the section “data collection” is appropriate.

**Results**

**15. Be mindful of the tense when reporting the results.**

**Response:** Thank you for pointing this out. The results section has been shortened and more detailed quotations are described in Appendix S4. The tense has been adjusted accordingly.

**16. Table 2.: No need to present both men and women. Table should be better presented. Need to mention what these numbers are somewhere e.g. n(%). Also add explanation of what these 'n' are since you will be also adding the n(%) so readers might be confused. Or present this better.**

**Response:** Thank you very much. You are absolutely right that the Table 2 is confusing. We modified the Table 2 and hope it now presents the data in a clearer way.

**Table 2.** Participant characteristics (n = 36).

|  | **general**  **practitioners** | **pharmacists** | **nurses** | **patients** | **total** |
| --- | --- | --- | --- | --- | --- |
| *n* | 8 | 8 | 6 | 14 | 36 |
| 1. age | 47.5 (10.0) | 44.14 (9.6) | 39.5 (13.4) | 57.8 (14.2) | 50.1 (13.8) |
| 2. age range | 30 - 57 | 34 - 61 | 26 - 58 | 23 - 75 | 23 - 75 |
| 3. women (%) | 37.5 | 25 | 50 | 57.1 | 44.4 |
| 4. drone  experience (%) | 100 | 14.3 | 0 | 7.1 | 16.7 |
| 4.1 number of  answers | *n* = 3 | *n* = 7 | *n* = 6 | *n* = 14 | *n* = 30 |
| 5. medication app  experience (%) | 75 | 85.7 | 16.7 | 0 | 32.2 |
| 5.1 number of  answers | *n* = 4 | *n* = 7 | *n* = 6 | *n* = 14 | *n* = 31 |

Note: Numbers in brackets indicate standard deviation.

**17. Please attribute all quotes properly - similarly for the rest of the results.**

**Response:** Thank you for pointing this out. Important example quotations have been retained in the text. More in-depth quotations were taken up in Appendix S4 and supplemented by the assignment of the interviewees (general practitioner, pharmacist, nurse, and patient).

**References**

Davis, Fred D. (1989): Perceived Usefulness, Perceived Ease of Use, and User Acceptance of Information Technology. In: *MIS Quarterly* 13 (3), S. 319. DOI: 10.2307/249008.

Elo, Satu; Kyngäs, Helvi (2008): The qualitative content analysis process. In: *Journal of advanced nursing* 62 (1), S. 107–115. DOI: 10.1111/j.1365-2648.2007.04569.x.

Helfferich, Cornelia (2011): Die Qualität qualitativer Daten. Manual für die Durchführung qualitativer Interviews. 4. Auflage. Wiesbaden: VS Verlag für Sozialwissenschaften / Springer Fachmedien Wiesbaden GmbH Wiesbaden.

Kothgassner, O. D.; Felnhofer, A.; Hauk, N.; Kastenhofer, E.; Gomm, J.; Ryspin-Exner, I. (2012): TUI: Technology Usage Inventory: Vienna: FFG.

Rejeb, Abderahman; Rejeb, Karim; Simske, Steve; Treiblmaier, Horst (2021): Humanitarian Drones: A Review and Research Agenda. In: *Internet of Things* 16, S. 100434. DOI: 10.1016/j.iot.2021.100434.

Rothgeb, Jennifer M.; Willis, Gordon; Forsyth, Barbara (2007): Questionnaire Pretesting Methods. Do Different Techniques and Different Organizations Produce Similar Results? In: *Bulletin de méthodologie sociologique. Bulletin of sociological methodology* (96), S. 5–31. Online verfügbar unter http://journals.openedition.org/bms/pdf/348.
