# Supplementary material for: “Of course, drones delivering urgent medicines are necessary. But I would not use them until…” Insights from a qualitative study on users’ needs and requirements regarding the use of medical drones
Source: PLoS One. 2023 May 8;18(5):e0285393. doi: 10.1371/journal.pone.0285393 (PMC10166545; doi:10.1371/journal.pone.0285393)
Supplement: Attachment — Submitted filename: responses_to_reviewers_2_PONE-D-22-13044.docx [file pone.0285393.s007.docx]

**Responses to reviewers**

Manuscript Number: PONE-D-22-13044

“Of course, drones delivering urgent medicines are necessary. But I would not use them until…” Insights from a qualitative study on users’ needs and requirements regarding the use of medical drones

PLOS ONE

Dear Reviewer,

Thank you very much for the opportunity to submit a revised version of our manuscript. We are very thankful for the constructive comments provided by the reviewers. In the following, we address all reviewers´ comments in a point-by-point manner and hope the manuscript is now ready for publication in PLOS ONE. Changed paragraphs were marked in green color throughout the manuscript.

Thank you very much.

Yours sincerely,

Denny Paulicke (corresponding author), on behalf of all co-authors

**Editor´s comments**

**When submitting your revision, we need you to address these additional requirements.**

**1. Please review your reference list to ensure that it is complete and correct. If you have cited papers that have been retracted, please include the rationale for doing so in the manuscript text, or remove these references and replace them with relevant current references. Any changes to the reference list should be mentioned in the rebuttal letter that accompanies your revised manuscript. If you need to cite a retracted article, indicate the article’s retracted status in the References list and also include a citation and full reference for the retraction notice.**

**Response:** Thank you for the important recommendation. Transmission errors of the literature management program had crept in, which we have corrected and marked. We have checked the reference list and the manuscript. It is now correct and complete.

**Reviewer 1,2 and 3**

**Comments to the Author**

**1. If the authors have adequately addressed your comments raised in a previous round of review and you feel that this manuscript is now acceptable for publication, you may indicate that here to bypass the “Comments to the Author” section, enter your conflict of interest statement in the “Confidential to Editor” section, and submit your "Accept" recommendation.**

Reviewer #2: (No Response)

Reviewer #3: All comments have been addressed

**2. Is the manuscript technically sound, and do the data support the conclusions?

The manuscript must describe a technically sound piece of scientific research with data that supports the conclusions. Experiments must have been conducted rigorously, with appropriate controls, replication, and sample sizes. The conclusions must be drawn appropriately based on the data presented.**

Reviewer #2: Yes

Reviewer #3: Yes

**3. Has the statistical analysis been performed appropriately and rigorously?**

Reviewer #2: Yes

Reviewer #3: Yes

**4. Have the authors made all data underlying the findings in their manuscript fully available?

The** [**PLOS Data policy**](http://www.plosone.org/static/policies.action#sharing) **requires authors to make all data underlying the findings described in their manuscript fully available without restriction, with rare exception (please refer to the Data Availability Statement in the manuscript PDF file). The data should be provided as part of the manuscript or its supporting information, or deposited to a public repository. For example, in addition to summary statistics, the data points behind means, medians and variance measures should be available. If there are restrictions on publicly sharing data—e.g. participant privacy or use of data from a third party—those must be specified.**

Reviewer #2: Yes

Reviewer #3: Yes

**5. Is the manuscript presented in an intelligible fashion and written in standard English?

PLOS ONE does not copyedit accepted manuscripts, so the language in submitted articles must be clear, correct, and unambiguous. Any typographical or grammatical errors should be corrected at revision, so please note any specific errors here.**

Reviewer #2: Yes

Reviewer #3: Yes

**6. Review Comments to the Author

Please use the space provided to explain your answers to the questions above. You may also include additional comments for the author, including concerns about dual publication, research ethics, or publication ethics. (Please upload your review as an attachment if it exceeds 20,000 characters)**

**Reviewer #2: Dear Authors, congratulations! After a long analysis, it was observed that all requested corrections were successfully met. From this perspective, the present article is approved to be published in this excellent journal. Grateful.**

**Response:** Thank you very much for the feedback and especially for the very valuable advice you gave us for the revision of the manuscript.

**Reviewer #3: A manuscript with a very attractive title. Abstract very well designed. The work in the introduction lacks information about what is novelty in the work, what gap it fills. In the research part, the authors imprecisely describe the participants in the research. This section needs to be refined, lines 118-130. The authors write that the participants were selected in the process but are not definitively characterized. It is also not known how many there were or on what basis the selection was made. The literature review in the work is quite poor and requires refinement. The conclusion also needs some fine-tuning.**

**Response:** Thank you for your feedback and for the laudatory assessment of the title and the abstract. The necessary adjustments and completed information have been added. The novelty of the work is emphasized at the beginning (lines 87-89). The present study was preceded by a systematic literature review (scoping review), which confirmed the research gap and led us to focus more strongly on the participatory nature of the research process. We have mentioned this again in the manuscript in lines 103-106.

We have specifically integrated the description of the participants into the results section in order to achieve a better comprehensibility of the results. From line 203 onwards, as well as in Table 2, the sociographic data and the characteristics of the participants are presented and described in detail. We have added this note in lines 134-135.

We have improved the structure of the conclusion and supplemented it once again with regard to the added value.

**7. PLOS authors have the option to publish the peer review history of their article (**[**what does this mean?**](https://journals.plos.org/plosone/s/editorial-and-peer-review-process#loc-peer-review-history)**). If published, this will include your full peer review and any attached files.**

If you choose “no”, your identity will remain anonymous but your review may still be made public.

**Do you want your identity to be public for this peer review?** For information about this choice, including consent withdrawal, please see our [Privacy Policy](https://www.plos.org/privacy-policy).

Reviewer #2: **Yes:** Gabriel Gomes de Oliveira

Reviewer #3: No

**Response:** Thank you very much for your very valuable feedback.
